# Removal of Cr (VI) from wastewater using bentonite as adsorbent: Experimental and Machine Learning investigations

**DOI:** 10.1038/s41598-026-52541-4

**Published:** 2026-05-13

**Authors:** Suman Pawar, Chikmagalur Raju Girish, Thomas Theodore, Asha Gowda Karegowda, Swathi Nayak

**Affiliations:** 1 Department of Chemical Engineering, Siddaganga Institute of Technology, Tumakuru, 572103 Karnataka India; 2 Manipal Institute of Technology, Manipal Academy of Higher Education, Manipal, India; 3 School of Chemical Engineering, Vellore Institute of Technology, Vellore, 632014 Tamil Nadu India; 4 Department of Master of Computer Applications, Siddaganga Institute of Technology, Tumakuru, 572103 Karnataka India

**Keywords:** Bentonite, Cr (VI), Adsorption, Optimization, Machine learning, Chemistry, Engineering, Environmental sciences, Materials science

## Abstract

Bentonite material was prepared and examined for the adsorptive removal of Cr (VI) from an aqueous solution. The bentonite was characterized by XRD, FTIR, SEM-EDS, BET and DLS analyses. The experimental design optimization results for the adsorption (AN) of Cr (VI) onto the bentonite adsorbent (AB) determined using the Central Composite Design were pH: 4, adsorbent dose (AD): 1.0 g/100mL, initial Cr (VI) concentration (ICC): 200 mg/L, and contact time (CT): 60 min for a maximum Cr (VI) removal of 86.92%. The Cr (VI) AN onto bentonite fitted to Langmuir isotherm and an AN capacity of 25.17 mg/g was obtained. The kinetics of the process showed Elovich model and the AB also exhibited good reusability over various cycles. Various machine learning methods such as Linear Regression (LR), random forest regressor (RFR), Support Vector Regression (SVR), Gradient Boosting (GB), Artificial Neural Networks (ANNs) were investigated. Among these, the GB and SVR come out as the best model achieved R^2^ (0.987, and 0.995) the lowest MAE (0.194, and 0.210) and RMSE (0.359 and 0.371), which predicts the AN value very close to the experimental results. This integration of machine learning and environmental chemistry provides a powerful tool for developing cost-effective and sustainable water treatment systems using naturally available materials.

**Supplementary Information:**

The online version contains supplementary material available at 10.1038/s41598-026-52541-4.

## Introduction

Water is one of the important needs of human being and is required for different industrial activities. Groundwater contamination due to heavy metals needs usage of sustainable remediation technologies for removing pollutants from the environment. Heavy metals discharge in wastewater leads to harmful effects on aquatic life and natural waters becomes it is unsuitable for drinking purpose^1^. The World Health Organization (WHO) has determined that the concentration of metal ions in water should be maintained between 0.01 and 1 ppm. However, current levels of heavy metal ions in effluents can reach as high as 450 ppm^2^. Among the several heavy metals that cause water pollution, chromium is the most harmful contaminants, characterized by its diverse sources and hazardous effects. As per the guidelines from World Health Organization (WHO), the chromium content in drinking water must be within 0.05 mg/L and 0.25 mg/L in wastewater^3,4^. It is normally found and mined primarily as chromite (FeCr_2_O_4_) and, to a lesser degree, as crocoite (PbCrO_4_) or chromic oxide (Cr_2_O_3_). Chromite is mainly utilized for the generation of chromium, its content varies in the range 40–55%^5^.

Chromium ions predominantly exist in two principal valence states: Cr (III) and Cr (VI), each characterized by distinct biochemical properties. Among these, hexavalent chromium (Cr (VI)) presents more significant ecological hazards^6^. Hexavalent chromium is generally present in the forms chromate (CrO_4_^2-^)_2_ and dichromate (Cr_2_O_7_^2-^) (, has relatively high levels of toxicity compared to other valence states due to its higher mobility, reduced biodegradability, and superior oxidizing potential and also it is carcinogenic and has mutagenicity^7^. It is utilized in various sectors of the chemical production industry, including petroleum, fertilizers, pesticides, pulp and paper, paints, dyes and pigments and plastics. It is also prevalent in the mining and metallurgy industry, as well as in the electroplating industry, which encompasses automotive, electronics, and aerospace applications^2^. Due to the excess use of this metal, its concentration is found to vary from 30-200 mg/L^8^. The chromite deposits have also been identified in several regions across various states such as Orissa, Karnataka, Maharashtra, Jharkhand, Andhra Pradesh and some parts of Tamil Nadu^9^. It is essential to effectively reduce the concentration of Cr (VI) from wastewater for maintaining water quality^10^.

There are several methods like chemical reduction, ion exchange, precipitation, electrochemical treatment, reverse osmosis, membrane separation, ultrafiltration and AN for treating wastewater. Among them AN is the one which is cost effective, simple and environmentally friendly^11^. To achieve an economically effective treatment of wastewater having heavy metals, different low-cost materials and commercially available materials have been investigated worldwide. They include Biochar^12^, metal based nanoparticle^13,14^. composite like chitosan-clay^15^, natural materials like fish shell powder^16^, activated carbon^17^. Bentonite is a natural, low-cost clay mineral and it is available abundantly. Through the removal of pollutants from wastewater by AB supports SDG 6 (i.e. Clean water and sanitation by rendering it safe for discharge and reusable). The use of bentonite prevents the release of toxic pollutants into water bodies thus protecting the aquatic life and biodiversity and supporting SDG 14 (i.e. Life below water which contributes to ecological balance). Hence, in this study bentonite was used as AB for the removal of Cr (VI) from wastewater by considering the various parameters like pH, time, temperature and AD by using response surface methodology (RSM).

AN is one of the effective and simple method for the treatment of heavy metals present in wastewater^18^. It does not depend high end technology and also on the high energy requirement for running the experiments. The AB is generally locally available and prepared from inexpensive chemicals, because of which it draws the attention in wastewater treatment^19^. Various analysts have examined the effectiveness of different AB’s for the removing Cr (VI) from wastewater in batch, fixed bed, and fluidised bed processes^20^. Also, because of raising awareness about global warming, it is required to use naturally available bentonite for the treatment of heavy metals from wastewater. Hence, in the present study, an attempt is to remove Cr (VI) from wastewater using bentonite and optimizing best conditions to industry effluent treatment.

As an interdisciplinary field interconnecting multiple researchers now keenly utilize machine learning (ML) in AN studies^21^. ML methods, such as GB, SVR, ANNs, RFR, and LR have gradually gained importance in the study of pollutant AN processes^22^. ML systems offer the additional benefit of optimizing AN parameters^23^ and outpacing traditional regression systems used for single or multi-component systems^24^. ML have features such as ANNs, SVM, RFR, and similar methodologies and have emerged as key tools in the study of organic pollutant AN^25^. These ML techniques serve dual roles: they optimize AN parameters^26^ and improve traditional regression approaches in modelling of AN scenarios^27^. AI algorithms have confirmed extraordinary competences in evaluating AN capacities, optimizing process parameters, and exploring relationships between variables which is helpful for experimental processes^28^.

The originality of this work lies in the usage of bentonite as an adsorbent for the removal of Cr (VI) from wastewater. The various characterization was done to know the properties and that helps in the determining AN mechanism. The optimization of parameters using RSM and finding the significant parameters which affects the AN. It involves checking the best operating parameters by using ML approach. The work also evaluates the maximum adsorption capacity from which it can be scaled up to the environmental remediation applications.

## Materials and methods

### Materials

Bentonite was procured from Heilen Biopharm Private Limited, Vadodara, Gujarat, India. Potassium dichromate ($\:{K}_{2}{Cr}_{2}{O}_{7}$), Sulphuric acid ($\:{H}_{2}S{O}_{4}$), nitric acid ($\:HN{O}_{3}$) and sodium hydroxide (NaOH) were obtained from E. Merck India Ltd. All reagents and standards were prepared using double-distilled water. All glassware was cleaned by soaking in 15% nitric acid and then thoroughly washed with distilled water before use.

### Equipment

Different equipment used for the AB preparation and for performing AN experiment are Hot air oven (Murhopye Scientific Company, India), orbital shaking incubators (Remi CIS-24 Plus, India), UV–Vis Spectrophotometer (Shimadzu-UV 1800-Japan). The characterization was done to study the various properties of the prepared AB.

The Brunauer–Emmett–Teller (BET) method was used to determine surface area and pore volume using Micromeritics, 3Flex Chemi, Norcross, GA. X-ray diffraction (XRD) analysis was performed using Rigaku, Model: SmartLab, Japan to study the amorphous nature of the material. The morphology of the AB before and after AN was investigated using a scanning electron microscope (SEM), TESCAN, Czech Republic. The Energy Dispersive X-ray Spectroscopy (EDS) analysis is carried out to determine the elemental composition of the AB. The functional groups on the surface of the ABs were identified suing Fourier Transform Infrared spectroscopy (FTIR), Perkin Elmer, United States. Dynamic Light Scattering (DLS) was used to find the particle size of the AB using Malvern, United Kingdom.

### Batch AN study

**Fig. 1:**
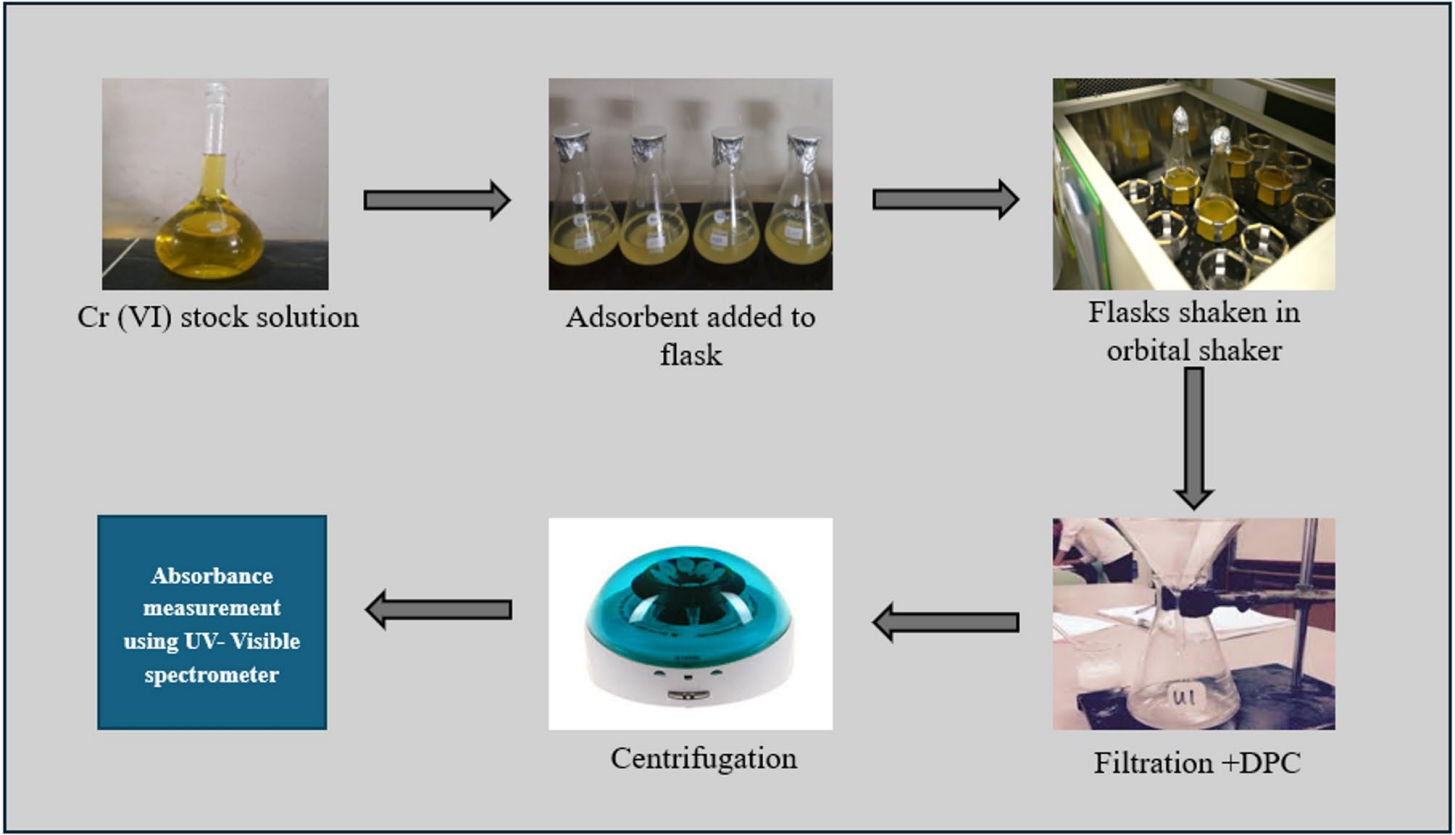
The pictorial representation of experimental setup for batch studies.

The bentonite was dried in an oven at 80 °C for 8 h and was used as an AB. A required amount of solution of Cr (VI) was prepared by required amount of K_2_Cr_2_O_7_ in distilled water. This solution was diluted to in the range of 50 to 200 mg/L. The pH of the solution was adjusted to the required value using NaOH or H_2_SO_4_. The AN test was carried out using 250 mL flasks containing known concentration of Cr (VI) to which a known mass (m) of AB was added. These flasks were agitated with a known volume of pollutant (V) on orbital shaker at 120 rpm and kept at 30 °C for 2 h^29^. After filtration the solution was centrifuged using 5000 rpm. The optical density was measured using UV–vis spectrophotometer at a wavelength of 540 nm. A schematic representation of the batch AN study and the estimating Cr (VI) concentration in the sample presented in Fig. 1.

The removal of Cr (VI) ions (%R) was determined by Eq. (1).

1 $$\:\mathrm{\%}\:\mathrm{R}\:=\:\frac{({\mathrm{C}}_{\mathrm{i}}-{\mathrm{C}}_{\mathrm{f}})}{{\mathrm{C}}_{\mathrm{i}}}\times\:100$$

where C_i_ and C_f_ are the initial and final Cr (VI) concentrations (mg/L), V is the volume of adsorbate (L), and m is the weight of the AB in (g).

### Optimization study

This study will be performed using Design Expert^®^ software (Version 9.0.3.1, Stat-Ease Inc., Minneapolis, MN) and was employed for optimizing the AN process. It was also used to understand the interaction belongings of each variables. The various levels of different parameters used for AN studies and its experimental range and levels are shown in Table S1.

#### Isotherm experiments

In this part trials were carried out in standard flasks containing ICC of Cr (VI) of 200 mg/L. The AD used was 1 g/100 mL, the pH was adjusted and held at 1.0. These flasks were kept of shaking at a speed of 120 rpm at 30 °C for 2 h.

#### Kinetics study

1 g of the AB was taken in a flask containing a 100 ml of Cr (VI) solution of ICC of 200 mg/L. The pH was adjusted to the required value and the flask was placed in a rotating shaker at 120 rpm and 30 °C. The sample was collected at consistent intervals of time during the experiment. The sample was filtered and then the Cr (VI) concentration was measured.

#### Desorption studies

The Cr (VI) adsorbed bentonite is mixed with 0.25 M HCl in 250 mL Erlenmeyer flask for 3 h in a shaker at 30 °C and washed with distilled water. The bentonite is set to settle and removed using a filter this is desiccated in the hot air oven at 50 °C until they get dried. The dried bentonite is used again for the desorption studies.

### Machine learning studies

#### Data collection and pre-processing

The raw dataset considered by experimental results to predict the AN property of bentonite, and the total number of datasets used was 100 points. The input variables of the model were the pH, CT, AD and ICC. Among the input variables, the data type were numerical for models to use as training materials using the one-hot encoding technique. For training and validation of the ML data, the 100 datasets were arbitrarily split into 70% and 30%, and the data values were regularized to have values between 0 and 1 through the min-max function so that the ML model could learn on a communal scale.

#### Model optimization

In this study, 4 models ANNs, RFR, and SVR were developed with Radial basis function (RBF) kernel using Python, and the predictive performance was matched with traditional LR to predict the AN property of bentonite. The ML work is carried out using Kaggle environment with Python (version 3.10). ML models are provided by Scikit-learn (version 1.2+) package. Scatter plots, bar charts, Ramp plots and feature selection by SHAP analysis are plotted using functions provided in Seaborn (version 0.12+) and Matplotlib (version 3.7+) packages. Table 1 summarizes the hyperparameters and their tested ranges used in pollutant removal prediction study for ANNs, RFR, SVR and GB.

**Table 1:** Summary of hyper-parameters and values ranges models used.

Model used	Hyper-parameter	Values used
ANNs	Hidden layers	1, 2
	Neurons per layer	32, 64
	Activation function used to introduce nonlinearity	relu, tanh
	Optimization algorithm	Adam
	Max iterations	1000, 2000
	Alpha (Regularization)	0.0001, 0.001, 0.01
	Learning rate	0.001, 0.01
RFR	Number of Trees	100, 200
	max_depth	3, 5, None
	min_samples_split (Spit control)	2, 5
	min_samples_leaf ( Leaf Stability )	1, 2
	Max_features for feature selection	sqrt, log2
SVR	Kernel (regression surface)	rbf, linear
	C (Regularization strength)	0.1, 1, 10
	Epsilon	0.01, 0.1
	Gamma	scale, auto
GB	n_estimators (Boosting stages)	100, 200
	learning_rate	0.05, 0.1
	max_depth (Tree depth)	2, 3
	Optimization loss	squared error

The performance of the models was evaluated based on the Root Mean Square Error (RMSE) , Mean Absolute Error (MAE) and coefficient of determination (***R***^**2**^) for the test data.RMSE is a value obtained by square root of averaging the sum of squares of the errors of the trial and the predicted value. It helps to understand the stability of the model. MAE provides information of how the predicted values differ from actual values on average. ***R***^**2**^ tells the information about how well the predictor variables can explain the variation in the response variable. ***R***^**2**^ lies in the range 0 to 1, with value of 1 signifying that model explains all variability perfectly. MAE and RMSE lies in the range 0 to positive value, with 0 indicating almost no variation between predicted and actual values. The RMSE, MAE and ***R***^**2**^ are computed using Eqs. (2)–(4)^30,31^.

2 $$\:\:RMSE=\surd\:\left[\left(1/n\right)\sum\:{\left({y}_{i}-{\widehat{y}}_{i}\right)}^{2}\right]$$

3 $$\:MAE=\left(\raisebox{1ex}{$1$}\!\left/\:\!\raisebox{-1ex}{$n$}\right.\right)\sum\:\left|{y}_{i}-{\widehat{y}}_{i}\right|$$

4 $$\:{R}^{2}=1-\left[\sum\:{\left({\boldsymbol{y}}_{\boldsymbol{i}}-{\widehat{\boldsymbol{y}}}_{\boldsymbol{i}}\right)}^{2}/\sum\:\sum\:{\left({\boldsymbol{y}}_{\boldsymbol{i}}-{\stackrel{-}{\boldsymbol{y}}}_{\boldsymbol{i}}\right)}^{2}\right]$$

where, y_i_ = Actual value, ŷ_i_ = Predicted value, $\bar{y}$ = Mean of actual values, n = Number of observations.

## Results and discussion

### Physicochemical properties of bentonite

Bentonite, a clay rock consists of primarily clay mineral montmorillonite. It has a composition of mainly SiO_2_, Al_2_O_3_, Fe_2_O_3_ and other minor components such as Na_2_O, CaO, TiO_2_, MgO, P_2_O_5_ and loss on ignition^32,33^.The properties of bentonite as obtained from BET analysis such as surface area, pore volume and pore diameter is given in the Table S2.

### Characterization of the prepared adsorbent

#### XRD

The characterization of bentonite samples by XRD is carried out to verify the presence of associated and clay minerals. Fig. S1 presents the XRD results of the bentonite sample. The XRD pattern shows the presence of montmorillonite at a 2θ degree of (6.1º, 23.2º, and 42.06º) and kaolinite (29.05º, 40.90º ,59.94º, and 62.5°) as major phase. The same results have been reported by^34^ The data shows the presence of impurities such as kaolinite (11.71º and 23.23º) and quartz (24.87º and 28.67º) in the bentonite used. Similar XRD results have been obtained in the research work ^35^. The obtained diffractogram compares well with the standard JCPDS No: 2-458, 3-863, 2-1227, 1-850.

#### FTIR

**Fig. 2:**
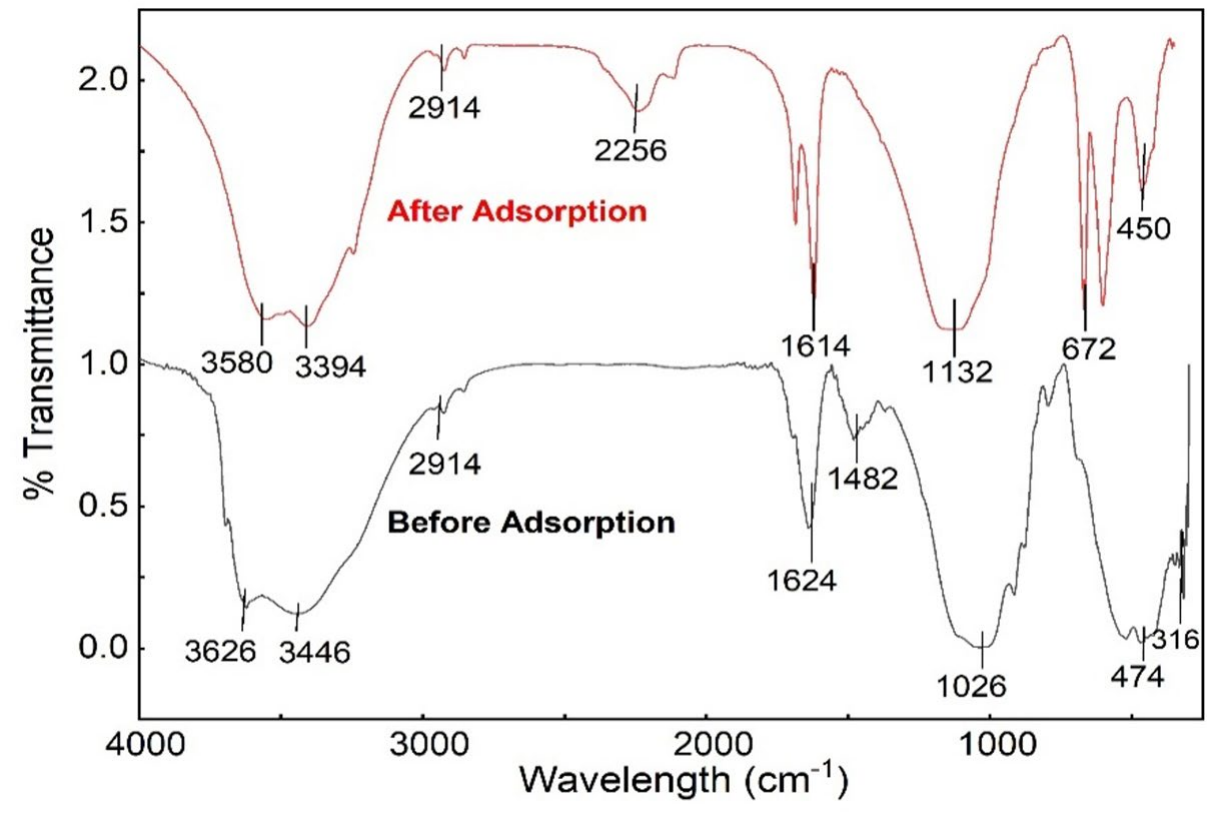
FTIR spectra of AB bentonite before and after AN.

The bentonite before and after Cr (VI) AN spectrum was performed in the array 4000 –400 cm^− 1^. The spectra and functional groups are shown in Fig. 2.

A peak at 3626 cm^− 1^ is due to vibrations of the OH^−^ groups for the H_2_O molecules adsorbed on to the bentonite surface. The bending vibration mode of water is indicated by a peak at 1614 cm^− 1^. These results have been corroborated by^36^. The band observed at 1026 cm^− 1^ is due to the stretching vibration of Si-O for silicate-based compounds. The peaks at 316 cm^− 1^ and 474 cm^− 1^ correspond to the bending vibrations of Al-O-Si and Si–O-Si. Similar results have been reported in the research^5^. The summits at 672 cm^− 1^ and 1132 cm^− 1^ (after AN) have slightly shifted from the peaks at 474 cm^− 1^ and 1026 cm^− 1^, respectively due to the contribution of Si-O-Al bending vibration and Si-O widening. The strength of many peaks has decreased. There is a decline in the intensity of the summits in the bands at 1624 cm^− 1^ and 1614 cm^− 1^ due to H-O-H bending. The bands at 316 cm^− 1^ and 474 cm^− 1^ correspond to bending vibrations of Al-O-Si and Si-O-Si bending. These peaks have disappeared probably due to the AN of Cr (VI) on to bentonite. Similar results have been reported by Ajemba^5^. Therefore, it is expected that the presence of Si-O-Al, Si-O-Si, carbonyl and hydroxyl groups might have influenced Cr (VI) AN on to bentonite. The above discussed peaks are represented in Table 2.

**Table 2:** Surface functional groups observed on the bentonite from FTIR.

Peak before AN, cm^− 1^	Peak after AN, cm^− 1^	Corresponding functional groups
316	450	Al-O-Si, Si-O-Si
474	672	Si-O-Si, Si-O-Al
1026	1132	Si-O, Si-O-Al bending
1482	1614	H-O-H bending
1624	2256	H-O-H bending
2914	2922	OH groups
3446, 3626	3394, 3580, 3410	OH groups

#### SEM and EDS

**Fig. 3:**
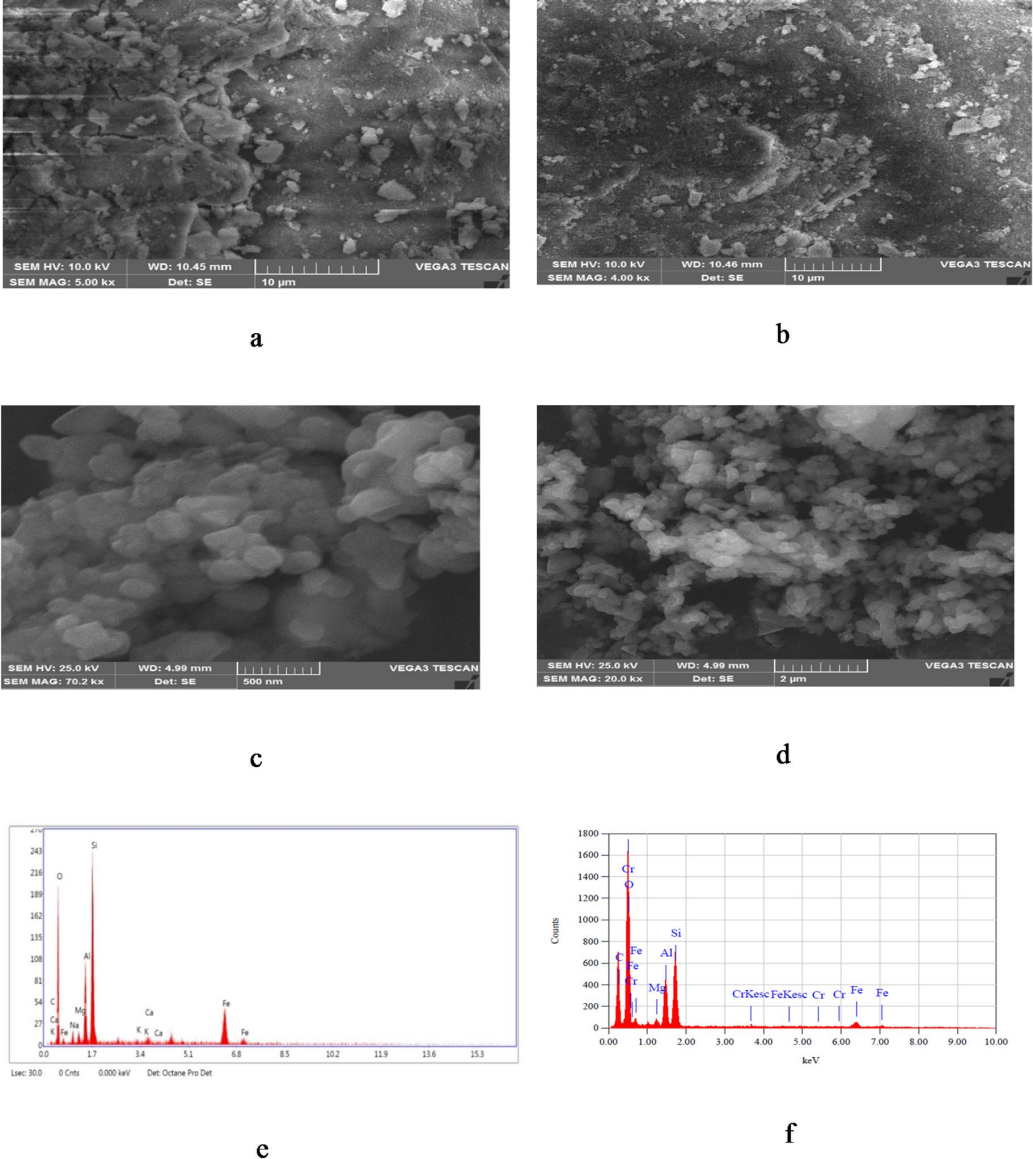
SEM-EDS images of Bentonite (**a**,**b**,**e**) before AN and (**c**,**d**,**f**) after AN.

SEM in combination with EDS was used to investigate the morphology of the AB particles and the percentage of the elements present in them. SEM images of the bentonite, before and after AN of Cr (VI) are shown in Fig. 3. The morphology of bentonite revealed flat surfaces with sponge-like structure (Fig. 3a,b) but after treatment with Cr (VI) solution, bentonite aggregates and Cr (VI) ions adhere to the surface of bentonite as presented in Fig. 3c,d. This could be due to the swelling of bentonite during the AN of Cr (VI). Thus, it can be concluded that in bentonite, the surface of the AB showed few particles of irregular shape adhering to the surface of the AB after AN whereas it is absent in the SEM images of the AB before AN^37^. The elemental composition of the bentonite, before and after AN was analysed by EDS and is depicted in Fig. 3e, f. The EDS study of bentonite revealed that the major constituents are Si (22.07%) and Fe (13.36%) by weight before AN. After Cr (VI) AN, elements like C, K, Na, and Ca were absent^38^.

#### DLS

DLS technique gives the information about the particle size distribution in a liquid system^39^. The DLS results showed in Fig. S2, and image showed that the Bentonite had an average size of 2177 nm, with PDI count 0.269 using Count Rate (kcps): 420.8.

### Optimization study

#### Optimization of bentonite by Central Composite Design (CCD)

The CCD was chosen for optimizing the bentonite. The ranges of the parameters selected according to Table 1. The experimental plan of Cr (VI) for bentonite reported in Table S3. The trials were done in duplicate and the average value has been reported.

5 $$\begin{aligned} \% {\text{ }}AN= & 144.15930--24.58297 \times pH+13.16442 \times AD - 0.29625 \\ & \times {\text{ }}ICC - 0.12038 \times CT - 0.57361 \times pH \times AD+0.011392{\text{ }} \\ & \times {\text{ }}pH \times ICC+0.014932 \times pH \times CT+0.033611 \times AD{\text{ }} \\ & \times {\text{ }}ICC+0.047525 \times AD{\text{ }} \times {\text{ }}CT+6.12424 \times {10^4} \times CT{\text{ }} \\ & \times {\text{ }}ICC+1.81175 \times p{H^2} - 7.12714 \times A{D^2}+1.14520 \times {10^{ - 3}} \\ & \times {\text{ }}ICC - 1.03465 \times {10^4} \times C{T^2} \\ \end{aligned}$$

The above regression model, Eq. (5) gives the relationship between the response and the independent variables in terms of the coded factor. It can be predicted from the equation that the dosage and solution pH have a positive effect on the AN and the initial Cr (VI) concentration has a negative effect on the AN capacity. The highest order of the significant effect by the different independent variables and their interactions on the AN capacity is ICC and the lowest is combined effect of AD and ICC.

*Analysis:* The analysis was done based on the percentage AN value. Various transformations were applied and different models as suggested by the software were employed. The model and deficiency of fit were considered for significance based on the p-values and the transformation with the highest R^2^ value was selected. Table 3 shows the analysis of variance (ANOVA) for the response of the elimination of Cr (VI) by bentonite.

**Table 3:** ANOVA analysis and its outcomes on AN.

Source	Sum of squares	Degree of freedom	Mean square	F value	*p*-value (prob > F)	Significance
Model	1841.72	14	131.55	86.69	< 0.0001	Significant (*)
A – pH	427.20	1	427.20	281.53	< 0.0001	*
B - AD	245.24	1	245.24	161.62	< 0.0001	*
C– ICC	738.43	1	738.43	486.64	< 0.0001	*
D – CT	34.36	1	34.36	22.65	0.0003	
AB	1.07	1	1.07	0.70	0.4151	
AC	11.68	1	11.68	7.70	0.0142	
AD	2.70	1	2.70	1.78	0.2023	
BC	20.59	1	20.59	13.57	0.0022	
BD	5.53	1	5.53	3.65	0.0755	
CD	25.53	1	25.53	16.82	0.0009	
A^2^	8.50	1	8.50	5.60	0.0318	
B^2^	5.40	1	5.40	3.56	0.0788	
C^2^	107.51	1	107.51	70.85	< 0.0001	
D^2^	0.016	1	0.016	0.010	0.9199	*
Residual	22.76	15	1.52			
Lack of Fit	19.08	10	1.91	2.59	0.1522	
Pure Error	3.68	5	0.74			
Rectified Total	1864.48	29				
Std. Dev	1.23		R^2^	0.9878		
Mean	66.12		Adj. R^2^	0.9764		
CV %	1.86		Pred. R^2^	0.9335		
			Adeq. Precision	37.676		

The F value of 86.69 shows the model is noteworthy. There was only 0.01% possibility of noise in the predicted model. The foretold R^2^ (0.9335) is in good covenant with the adjusted R^2^ (0.9764). The ratio of signal to noise (> 4.0) signpost acceptable model discernment^40^. In this study, acceptable value was found to be 33.456 which is (> 4.0) and considered satisfactory. The significance of the model data is shown by the F value of 86.69 and the p value of less than 0.0001. Based on the F data and p value, the ICC with F value of 486.64 and *p* < 0.0001 was found to be most significant factor. Also, other parameters such as AB dosage and pH were found to be significant factors. Similar type of results were found in the reported work^41–43^. Fig. S3 displays the 3-D response surface plots for various parameters. The points are clustered around the 45° line thereby signifying a moral treaty between the real and foretold standards.

#### Validation and confirmation of the model and contour plots

Validation is a technique of RSM that is used to check the accuracy of the predicted model and the corresponding results.

Fig S4a shows the validity of a model which can be evaluated by the residual analysis. It is the part which is not discussed by the equation of the model. This analysis can also identify some outliers among the total data. It generally represents some graphical methods for the residual analysis^44^. This is required to verify the homoscedasticity hypothesis of the errors.

The perturbation is plotted for all the various factors in Fig. S4b. Then, the influence of each factor is plotted at a definite point, while the other parameters are kept constant for Cr (VI) removal. The mid-point of two extreme values of every factor was selected as the reference point. AD, ICC, and temperature were found to be important following the center points^45^.

Fig S4c, revealed that the points are clustered around the 45° line thereby indicating a moral treaty between the real and foretold standards. In this study, the factors were within the range and based on the corresponding 10 optimum conditions, the AN capacities were about 86.92 mg/g. From 10 optimum conditions in the numerical optimization method, the desirability as an objective function was found to be 1 by considering the desired goals. Thus, the regression model successfully justified the process with high accuracy and is found to be practical for the prediction of AN capacity.

Further, Fig S4d shows the Box-Cox plot used to verify the *λ*-value, which is generally used to envisage the change in the experimental numerals to enhance the model impact. In accordance with the *λ*-value of 1.0 obtained from the plot, a natural log transformation was utilized. This plot is used to determine the most appropriate transfer function to apply to the specified responses. From the Box-Cox diagram, the best lambda value for bentonite was determined to be 1. This shows that the experimental data for the Cr (VI) treatment process should not be transferred to enhance the model and have sufficient accuracy^46^.

The combined effect of the dose, pH, CT, and ICC on Cr (VI) removal i.e. contour plots are shown in Fig. S5. The contour plot signifies that the AN capacity increases by increasing the pH from 5 to 4. But, it decreases by changing the pH from 5 to 6. At a pH less than 5, the bentonite surfaces become positively charged due to the higher H^+^ ion concentration, resulting in a lower AN capacity due to repulsion between the positively charged bentonite surface and Cr (VI). The solution favoured a higher AN capacity at pH 4 due to an electrostatic attraction between bentonite and Cr (VI). Hence, selection of an exact pH is very important and optimum pH was found to be 4. In this plot, researcher observed that the AN increased with increasing AD whereas, at the same time the AN percentage increased with increasing Cr (VI) concentration. However, a change in the AN capacity beyond 1 g/ L AD was insignificant. Therefore, the optimum dose was considered to be 1 g/L with the equivalent experimental conditions^47^.

### Influence of temperature

The effect of temperature is one of the most important factors in the AN process. The experiments were carried out at various temperatures. The effect of temperature on the AN of pollutant onto bentonite. The plot is depicted in Fig. S6. The fraction of Cr (VI) ions removed decreases as the temperature increased from 30 to 50 °C, by fixing pH 4, AD 1 g and agitation speed 200 ppm at 60 min. This is because of at high temperatures, few active sites were available and their surface area was significantly reduced. Thus, the equilibrium was established between the pollutant and AB components^48^. As a result, the current AN process is found to be exothermic. At 303 K, the removal efficiency of Cr (VI) ions was obtained as 87.5%.

### Effect of coexisting ions

The ionic strength effect was tested at room temperature and pH 4, by using NaCl of various concentrations of 0.1, 0.3, 0.5, 0.7 and 1 M as a background electrolyte. Fig. S7 shows the impact of ionic strength on the AN process. These figures clearly reveal that less pollutant was adsorbed by bentonite in the presence of competing cations (Na^+^) than when no cations were added; in addition, the efficiency of AN was progressively decreased with increasing ionic strength. This effect is usually interpreted as indicating a non-specific AN mechanism^49^.

### Isotherm study

**Fig. 4:**
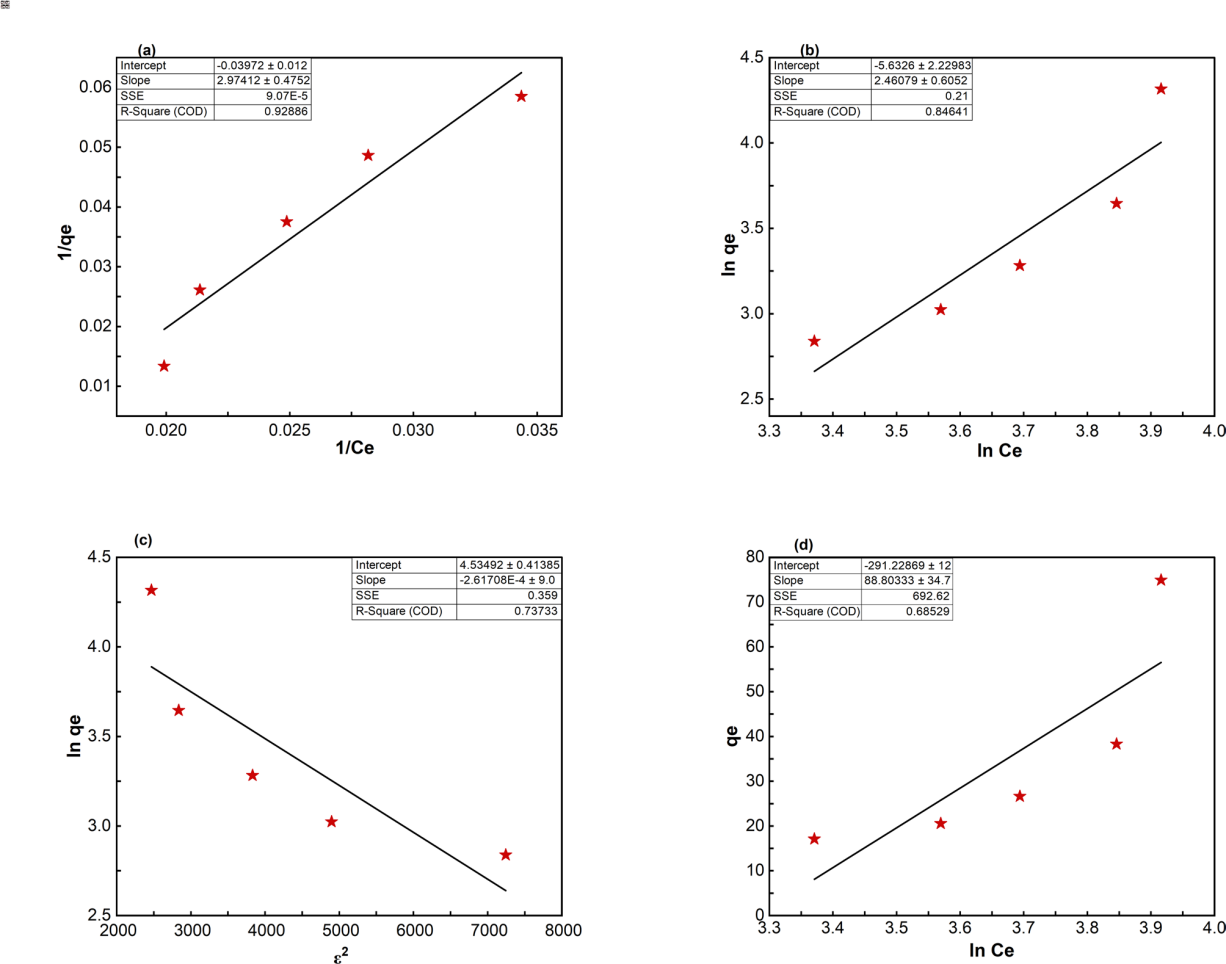
Validation isotherm models (**a**) Langmuir, (**b**) Freundlich, (**c**) D-R, and (**d**) Temkin isotherms.

The R^2^ (0.846) values of the Freundlich AN isotherm for bentonite shown in Fig. 4b, were found to be better value compared with the other isotherms. The slopes for bentonite, (*n* = 0.4) satisfies the ailment 0 < *n* < 1 and is a measure of the AN intensity^43^. A value (< unity) implies chemisorption^50^. Reciprocal of slope, 1/n above one for bentonite, (2.46) is indicative of cooperative AN. The data fitted the Freundlich AN isotherm well for bentonite. The values of K_L_ and q_max_ were found from the slope (S) and the intercept (I) of a linear plot of 1/q_e_ versus 1/C_e_ in Fig. 4a. The best fit of the Langmuir isotherm with a high R^2^ value shows the formation of a single layer on the exterior of the AB^51^. In this case, the R_L_ (0.27) values of the AB’s are between 0 and 1indicating that the AN is favourable and suggests monolayer^52^. For the D-R AN isotherm, ln q_e_ v/s ε^2^ gives β (mol^2^/kJ) and AN capacity, q_m_ (mg/g). The D-R isotherm parameters were calculated for the AB and presented in Table S4. The A_T_ and B_T_ of the Temkin model were obtained from the linear plot of a plot of qe v/s ln C_e_ (Fig. 4d). The R^2^ values of the D-R and Temkin plots were comparatively low for bentonite. Maximum AN capacity at the expenditure of minimum energy was obtained as q_max_ (25.17) and K_L_ (0.0133) for bentonite^53^.

### Kinetics study

**Fig. 5:**
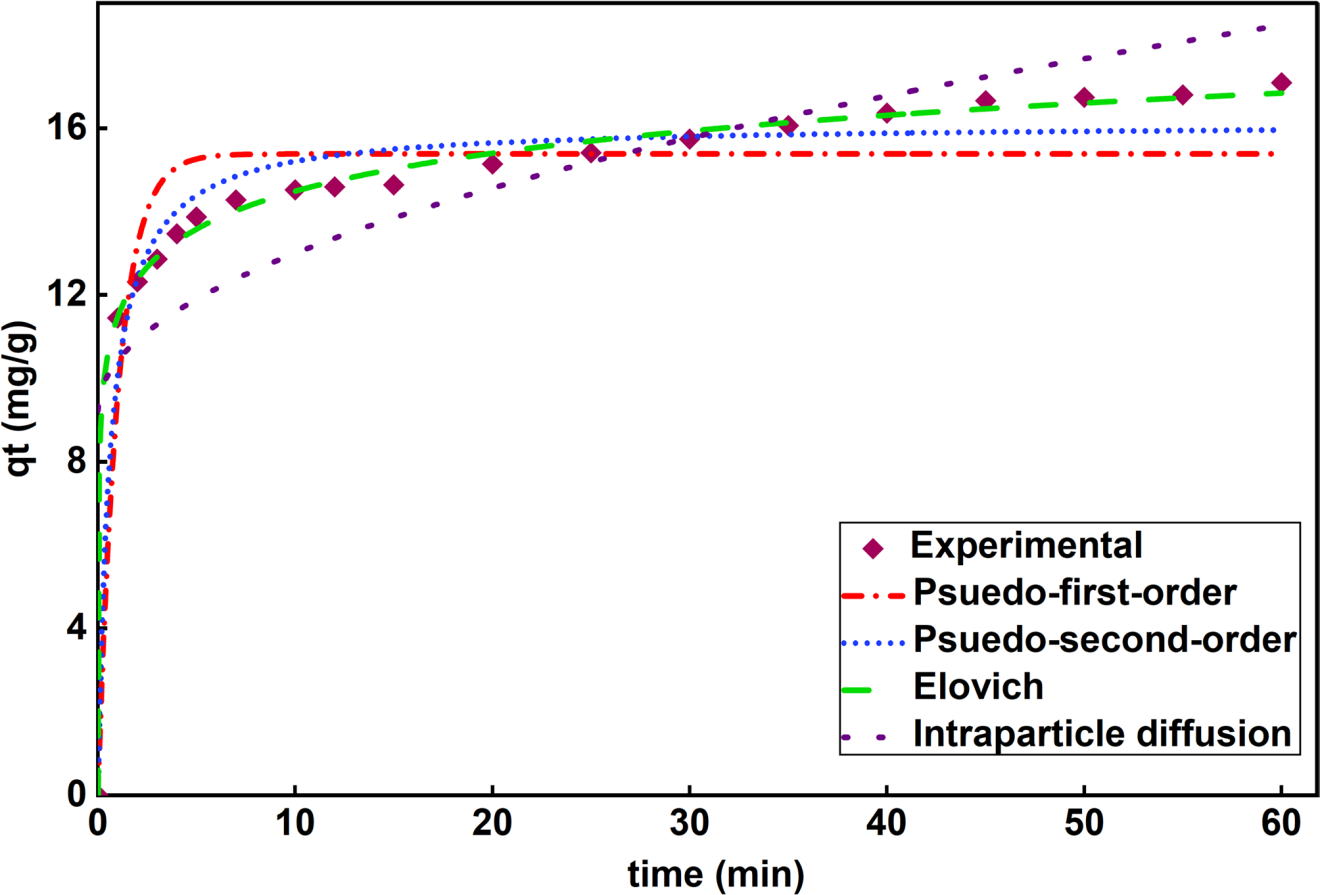
The validation of kinetic data to various models for the AN of Cr (VI).

Pseudo first order, pseudo second order, Elovich and intraparticle diffusion model were verified for the studying the AN of Cr (VI) removal by bentonite models and are shown in Fig. 5.

The constants of the various kinetic models obtained from the non-linear approach are presented in Table 4. A plot of $\:{q}_{t}$ v/s $\:t$ for various models are plotted and are compared with the experimental values. The goodness of fit was checked by$\:{R}^{2}$ and error function values. The experimental AN capacity was compared with the AN capacity obtained from the models. Based on the these values the data were fitting best with the Elovich model with $\:{R}^{2}$ value of 0.997 and lower error function values. From the pseudo-second order model the experimental value was matching with the model value, showing that rate-limiting step is because of chemical AN concerning valence forces through exchange of electrons between AB and the adsorbate^54^. From the Elovich constants, $\:\alpha\:$ suggests higher AN rate and $\:\beta\:$ suggests desorption linked activation energy and surface coverage. Therefore by comparing these values, it shows higher AN with minimum desorption^55,56^.

**Table 4:** The evaluation of kinetic parameters for the AN of Cr (VI).

Model	Parameters	Obtained values
	$\:{q}_{exp}$ (mg/g)	17.09
Pseudo-first order	k_1_ (min^− 1^)	0.969
	q_1_ (mg/g)	15.38
	R^2^	0.903
	χ^2^	1.457
	SSE	24.77
Pseudo-second order	k_2_ (g/mg.min)	0.103
	q_2_ (mg/g)	16.12
	R^2^	0.965
	χ^2^	0.517
	SSE	8.786
Intraparticle diffusion	k_id_ (mg/g.min^1/2^)	1.193
	C	9.225
	R^2^	0.565
	χ^2^	6.545
	SSE	111.2
Elovich	α (mg/g.min)	2.76
	β (g/mg)	0.761
	R^2^	0.997
	χ^2^	0.039
	SSE	0.673

### Reusability

The reusability has great importance to ensure the economics of using Bentonite many times. Fig. S8 displayed that the AN efficacy declines slightly in each successive step i.e., the drop from 86.92% in the 1st cycle to 48.2% in the 5th cycle, can be due to the occupying of some AB sites throughout these AN-desorption phases and also some AN sites are eventually occupied by Cr (VI) molecules^57^. Its reduction from 86.92% in the first cycle to 48.2% in the fifth cycle can be due to loss of some AB sites due to abrasion and also some AN sites were completely occupied^58^. Similar kind of results discussed by authors^59^.

### Mechanism

**Fig. 6:**
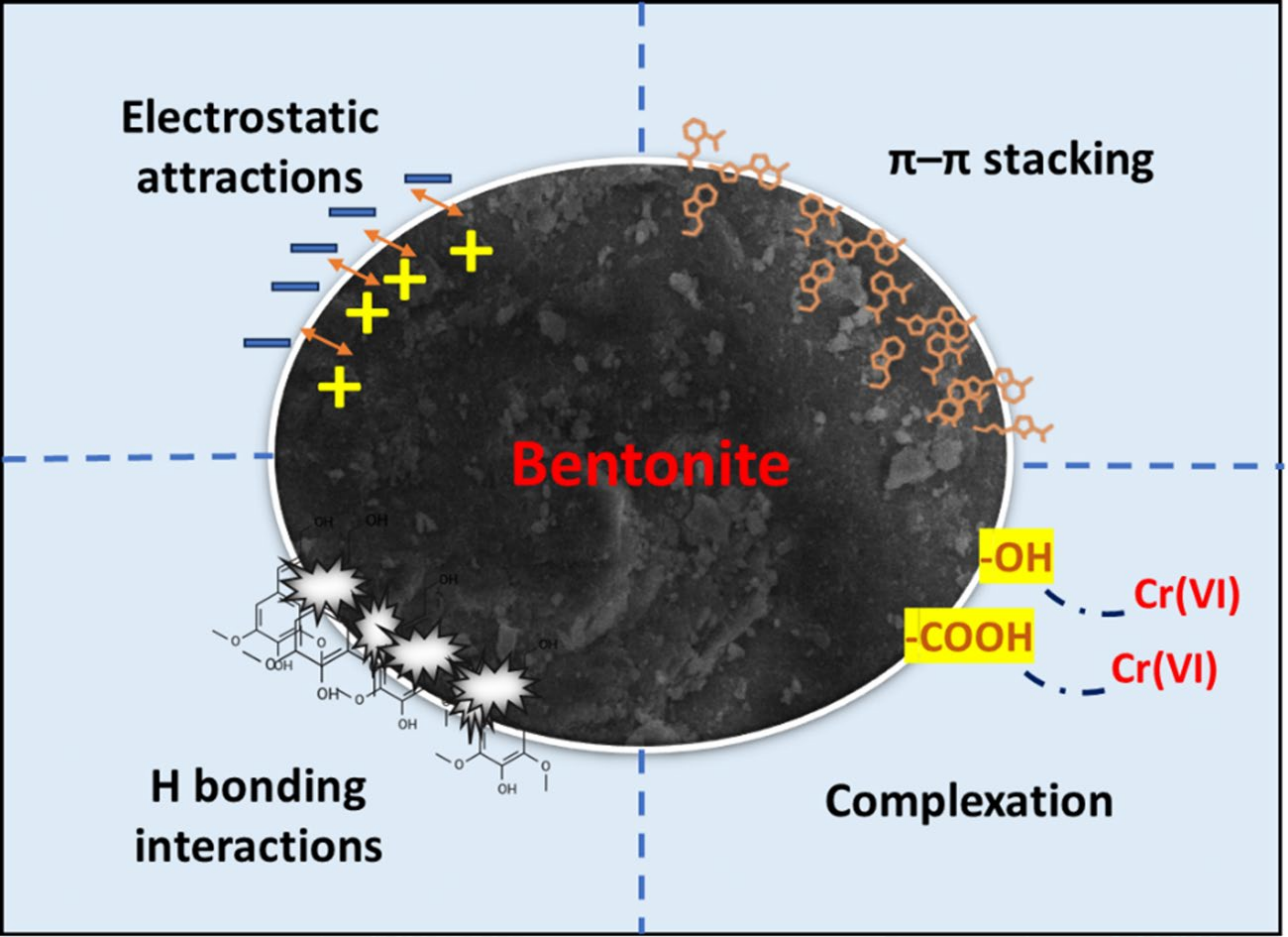
Schematic representation showing the interaction mechanisms between bentonite and Cr (VI).

The mechanism between bentonite and the Cr (VI) pollutant is illustrated in Fig. 6. These include electrostatic attraction, surface complexation, π–π interactions and Hydrogen bonding interactions. At lower pH values, the surface of the AB becomes positively charged due to protonation of functional groups. This interaction increases the electrostatic attraction between AB and negatively charged adsorbate. Also, hydroxyl groups interaction occurs between Cr (VI) and bentonite which stabilizes the adsorbed adsorbate and improve the removal efficiency. π–π stacking interactions contribute to additional stabilization due to if organic moieties or aromatic structures are present on the adsorbate surface. In addition, surface complexation plays a crucial role in Cr (VI) removal. The functional groups such as carboxyl, carbonyl and hydroxyl on the AB surface can easily bind with the adsorbate leading to the formation of inner or outer sphere structures^7,60^. To summarize, these mechanisms combinedly contribute to effective removal of Cr (VI).

### Result interpretation by machine learning

**Fig. 7:**
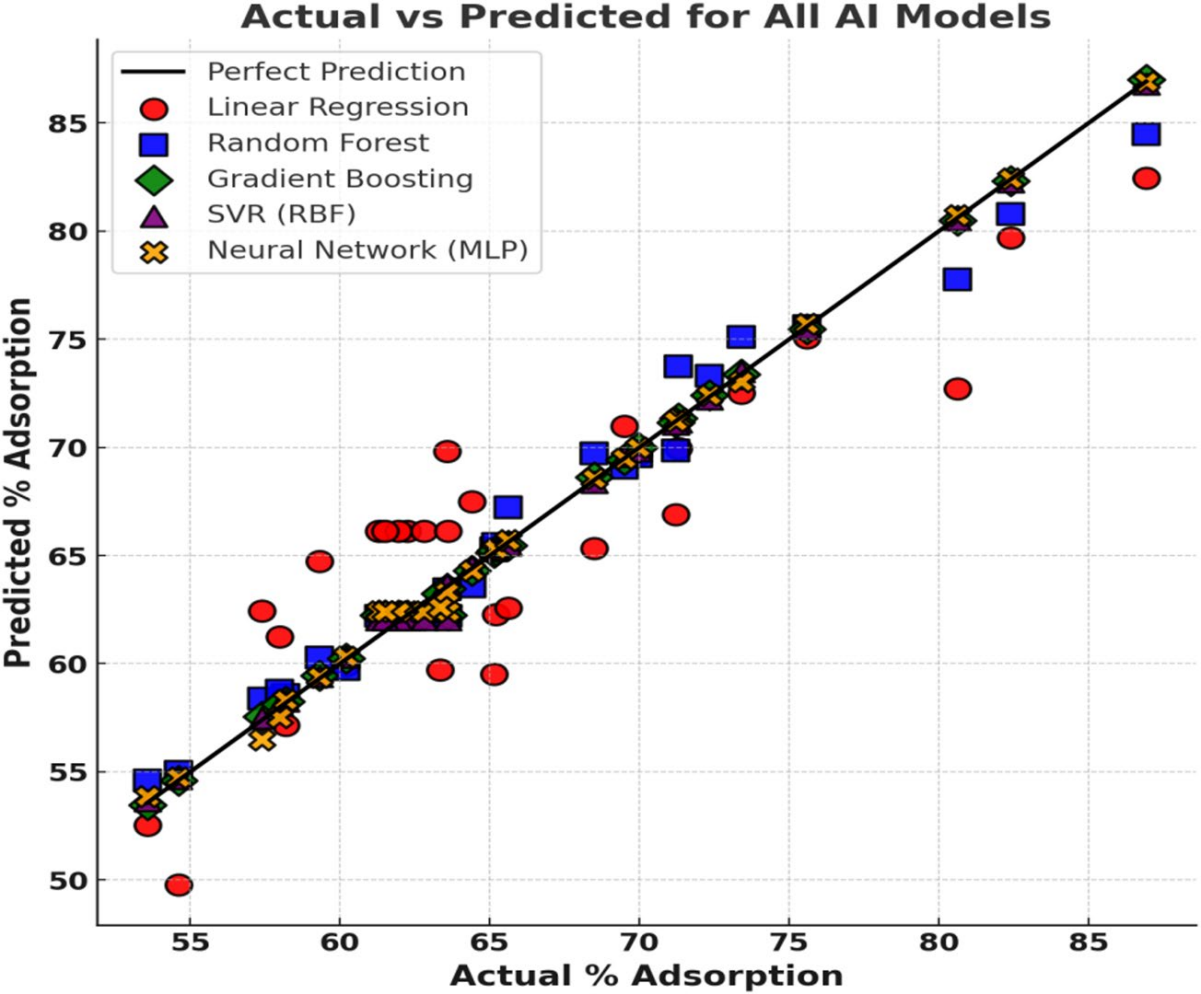
Comparative graph depicting the Actual v/s predicted of all 5 models for pollutant removal.

To analyse actual vs. predicted AN model a LR results were compared with ANNs, RF, GB, SVR(RBF) and ANNs. Individual plots of actual v/s predicted for LR, RF, GB, SVR(RBF) and ANN are depicted in Fig. S9a-e respectively. The comparative graph of actual vs. predicted results are shown in Fig. 7. Each model is represented distinctly without overlapping with other curves. This graph helps us visually compare how each model behaves when predicting percentage AN across the entire range of experimental conditions^61^.

The plot depicts GB and SVR show impressive prediction performance. Their prediction points lie almost exactly on the main pattern of the curve, showing that these two models understand the underlying AN behaviour much better than the others. Their points are tightly clustered around the accurate trend, with very few deviations. This is consistent with their high R² values and low error metrics. The performance of RFR and the ANNs appear in the middle zone. Their points follow the overall pattern, but they show slightly more spread and small deviations. They do not match the real trend as closely as GB and SVR, but they still perform reasonably well. Their behaviour in the graph shows that they capture the general direction of the AN but are not as precise in capturing the finer variations. On the other hand, LR clearly stands out from the rest with relatively poor performance. Its points deviate noticeably from the main curve, and it struggles to follow the nonlinear nature of the dataset. In the graph, the LR points look more scattered and misaligned when compared to the other models. This visual observation supports its weak performance in the statistical evaluation, where it had the lowest $\:{\boldsymbol{R}}^{2}$ and the highest error values^62,63^. Overall, the combined graph makes it very easy to understand how each model performs. The closeness of the points to the true trend shows the accuracy of each model. The more a model follows the natural pattern of the data, the better it is. From the visual comparison, GB is the most accurate, SVR is the second-best, RFR and ANNs show moderate performance, and LR performs the worst.

When researcher train a ML model to predict the percentage AN, one of the most useful outcomes understands which experimental factor is influencing the AN the most. This is called feature importance. It simply tells us how strongly each input variable (pH, dose, time, concentration) affects the predicted AN value.

When ML is trained to predict the percentage AN, one of the most useful outcomes understands which experimental factor is influencing the AN most. This is called feature importance. It simply tells us how strongly each input variable (pH, dose, time, concentration) affects the predicted AN value. The AN property of bentonite is affected by various factors, and the degree of influence varies reliant on each factor. Shapley Additive exPlanations (SHAP) analysis was achieved to analyse the contribution between variables towards AN property of bentonite. SHAP adopts the concept of Shapley value to analyse the involvement of input variables, which makes it easy to interpret ML models, comprehend prophecy logic, and verify the consistency and justice of the model. The SHAP analysis was performed using the SHAP library in Python 3.9.19^64^ with the best prediction model GB.

Figure S10 revels the SHAP standards related with the input features and prominence derived from a GB model used to predict the AN onto bentonite. The features are ranked in descendant order based on their average SHAP values, with those hierarchical uppermost exerting the most noteworthy influence on the model’s upshots. This scrutiny specifies that equilibrium concentration and particles’ specific surface area are the primary determinants of the AN degree of bentonite. These findings are critical for finding the key factors governing bentonite AN degree, providing appreciated perceptions for forthcoming research and optimization strategies. This lucidity improves model presentation and applicability in real-world scenarios, eventually permitting investors to make data-driven decisions by accepting the unique influence of each feature on the model’s predictions^18^.

Initial Concentration (D) turned out to be the most influential factor. The model places the highest importance on this parameter, which means that changes in the dye concentration strongly impact % AN. This perfectly matches ANOVA table, where D had the highest F-value (486.64) and was highly significant (*p* < 0.0001). pH is the next major contributing factor. The model identifies pH as a strong influencer because AN mechanism, surface charge, and dye ionization all vary with pH. The ANOVA table also shows a very high significance for pH with a large F-value (281.53) and *p* < 0.0001. AD plays a moderate role in affecting AN %. When more AB is present, more active sites are available, increasing removal efficiency. However, the model shows that dose is less influential than pH and concentration, which is again consistent with your ANOVA results (F-value = 161.62, *p* < 0.0001) CT is the variable with the smallest influence. Even though AN increase with time, your data shows that after a certain period (32–60 min), the process reaches a stage of saturation. This explains why the model gives CT the lowest importance. In the ANOVA table, time also had the smallest F-value among the main factors (22.65).

The performance of the ML models can be further better understood using $\:{R}^{2}$, MAE, and RMSE which helps to analyse how well a model matches the experimental AN value as shown in Fig. S11. In our case, $\:{\boldsymbol{R}}^{2}$ helps us understand whether the model is able to capture the behaviour of pH, AD, CT, and ICC on Cr (VI) removal. Fig. S11 shows, GB resulted in highest $\:{R}^{2}$ of 0.987 followed by acceptable performance of RFR, ANNs and SVR compared to LR ( $\:{R}^{2}$ of 0.842)^24^.

Figure S11 proves poor performance of LR model with MAE of 3.193 and lowest MAE of 0.194 with GB model. The other three model SVR, ANNs and RFR also prove to better with low MAE of 0.210, 0.665 and 0.937 respectively. Figure S11 proves poor performance of LR model with RMSE of 3.738 and lowest MAE of 0.738 with GB model. The other three model SVR, ANNs and RFR also prove to better with low RMSE of 0.371, 0.972 and 1.165 respectively.

Desirability ramp plots for multiple ML models were compared to analyze the influence of independent variables such as pH, AD, CT and ICC on dependent variable AN. GB and RF ramp plot showed step-like behaviour due to their tree-based structure and provided higher AN values. Contrarily LR exhibited linear trends, while SVR and MLP resulted in smoother but less accurate predictions. GB attained the uppermost AN of 86.97%, designating superior predictive and optimization competency^65^.

The desirability ramp plot for the best prediction model i.e. GB is depicted in Fig. S12a with each subplot representing the optimal value of one of the independent variables while keeping the rest of independent variables constant for maximizing the dependent variable AN. The perpendicular dotted line point to the optimal values of the independent variable. The corresponding anticipated AN is 86.97% with a desirability of 1, signifying the finest conceivable effective situation. The plot indicates the fall in the AN with the increase in the pH value, and 4.0 as optimal pH value. In similar lines the AN gradually increases with the increase of AD and CT with the optimal values of 0.78 and 46.72 respectively. The ICC does not show any variation in AN for the value ranging from 50 to 160, and there is sudden hike in AN for optimal value of ICC of 163.79. Overall, the low pH, high AD, moderate CT and high ICC results in maximum AN.

The ramp plot of SVR attained maximum AN of 67.49% with optimal values of pH, AD, CT and ICC as 4.0, 0.84, 58.10 and 200.0 respectively as illustrated in fig. S12b. The ramp plot of RF model in Fig. 12c indicates anticipated maximum AN is 84.63% with optimal values of pH, AD, CT and ICC as 4.0, 0.78, 46.72 and 163.79 respectively^66^.

The ramp plot of MLP model in fig. S12 (d) indicates anticipated maximum AN is 86.92% with optimal values of pH, AD, CT and ICC as 4.0, 1.0, 60.0 and 200.0 respectively. The ramp plot of LR model in fig. S12 (e) indicates anticipated maximum AN is 82.47% with optimal values of pH, AD, CT and ICC as 4.0, 1.0, 60.0 and 200.0 respectively^66^.

## Conclusion

This present study successfully utilized bentonite as an AB for the removing Cr (VI) from wastewater. The AN of Cr (VI) onto bentonite, was confirmed by the characterization by FT-IR and SEM-EDS to attain information about the composition, bonding, and morphology of the particles. From the CCD, based on F and p values, the factors were found to be significant. The ICC with F value of 486.64 and *p* < 0.0001 was found to be most significant factor. Other factors such as pH and AD were also found to be significant. The percentage AN for bentonite, was 86.92% at pH 4, AD 1 g/100mL, 200 mg/L ICC, and CT 60 min. The isotherm data fitted well with the Langmuir isotherm showing monolayer AN with an adsorption capacity of 25.17 mg/g. The kinetics followed Elovich model, showing that AN rate is more than the desorption rate. From the reusability studies, the adsorbent was found to be effective for AN till 5th cycle with a removal of 48.2%. The 3D response surface plots display the combined effects of the different parameters that influence the AN process. GB comes out as the best model with highest $\:{R}^{2}$ (0.987) and the lowest MAE (0.194) and RMSE (0.359), which means it predicts the AN value very close to the actual experimental results. It also handles the nonlinear behaviour of the AN process very well, making it the most reliable model for this study. The SVR performs almost as well as GB. Its errors are low, and its R^2^ is high, showing that it is also very accurate. From the SHAP analysis found for bentonite AN degree, Initial Concentration (D) turned out to be the most influential factor followed by pH as the next major contributing factor. Desirability ramp plots for multiple ML models were compared to analyse the influence of pH, AD, CT and ICC on AN. GB and RF ramp plot showed step-like behaviour due to their tree-based structure and provided higher AN value. Contrarily LR exhibited linear trends, while SVR and MLP resulted in smoother but less accurate predictions with LR. GB attained the uppermost AN of 86.97%, designating superior predictive and optimization competency. Therefore, it can be concluded that bentonite is a promising AB for removing the Cr (VI) from wastewater.

## Supplementary Information

Below is the link to the electronic supplementary material.

Supplementary Material 1

## Data Availability

The authors declare that the dataset supporting the findings is available within the article and its accompanying supplementary file. Further enquiries regarding the datasets generated during the current study can be directed to the corresponding author C.R.G.
