# Supplementary material for: Removal of Cr (VI) from wastewater using bentonite as adsorbent: Experimental and Machine Learning investigations
Source: Sci Rep. 2026 May 13;16:21926. doi: 10.1038/s41598-026-52541-4 (PMC13365516; doi:10.1038/s41598-026-52541-4)
Supplement: Supplementary file 1 — Supplementary Material 1 [file 41598_2026_52541_MOESM1_ESM.docx]

**Removal of Cr (VI) from wastewater using Bentonite as adsorbent: Experimental and Machine Learning investigations**

Suman Pawar ^1*^, Chikmagalur Raju Girish^2*^, Thomas Theodore^3^_,_ Asha Gowda Karegowda^4^, Swathi Nayak^2^

^1^Department of Chemical Engineering, Siddaganga Institute of Technology, Tumakuru – 572103, Karnataka, India

^2^Manipal Institute of Technology, Manipal Academy of Higher Education, Manipal, India

^3^School of Chemical Engineering, Vellore Institute of Technology, Vellore – 632014, Tamil Nadu, India

^4^Department of Master of Computer Applications, Siddaganga Institute of Technology, Tumakuru – 572103, Karnataka, India

*Corresponding authors E-mail :svp@sit.ac.in, cr.girish@manipal.edu

# **SUPPLEMENTARY FILE**

**List of Tables**

| **Table S1** | Factors and levels used for the CCD | 3 |
| --- | --- | --- |
| **Table S2** | The properties of bentonite obtained from BET analysis | 4 |
| **Table S3** | Experimental plan and the percentage Cr (VI) AN for bentonite | 4 |
| **Table S4** | The evaluation isotherm parameters for the removal of chromium | 5 |

**List of Figures**

| **Fig. S1** | XRD pattern obtained for the adsorbent bentonite | 8 |
| --- | --- | --- |
| **Fig. S2** | Particle size distribution of the Bentonite | 8 |
| **Fig. S3** | 3-D Response surface plots with respect to various parameters | 9 |
| **Fig. S4** | Diagnostic plots generated during ANOVA (a) Residual (b) perturbation (c) predicted vs actual (d) Box-Cox transformation plots | 10 |
| **Fig. S5** | Contour plots on effect of independent variables on the AN capacity | 11 |
| **Fig. S6** | The influence of temperature on removal of chromium. |  |
| **Fig. S7** | The effect of coexisting ions on the removal of chromium. |  |
| **Fig. S8** | Reusability of used bentonite over various cycles |  |
| **Fig. S9 (a)** | Actual v/s predicted values of LR model for pollutant removal study |  |
| **Fig. S9 (b)** | Actual v/s predicted of RF model for pollutant removal study |  |
| **Fig. S9 (c)** | Actual v/s predicted of GB model for pollutant removal |  |
| **Fig. S9 (d)** | Actual v/s predicted of SVR (RBF) model for pollutant removal study |  |
| **Fig. S9 (e)** | Actual v/s predicted of ANN model for pollutant removal |  |
| **Fig. S10** | Feature importance of SHAP analysis for Gradient Boosting |  |
| **Fig. S11** | $R^{2}$, MAE, and RMSE prediction models for AN property of bentonite |  |
| **Fig. S12 (a)** | Desirability Ramp plot for GB prediction models for AN property of bentonite |  |
| **Fig. S12 (b)** | Desirability Ramp plot for SVR prediction models for AN property of bentonite |  |
| **Fig. S12 (c)** | Desirability Ramp plot for RF prediction models for AN property of bentonite |  |
| **Fig. S12 (d)** | Desirability Ramp plot for RF prediction models for MLP property of bentonite |  |
| **Fig. S12 (e)** | Desirability Ramp plot for LR prediction models for AN property of bentonite |  |

**Table S1:** **Factors and levels used for the CCD**

|  | **Level code** | | |
| --- | --- | --- | --- |
| **Factor** | -1 | 0 | +1 |
| **ICC ( mg/L )** | 50 | 125 | 200 |
| **AD (g/100 mL)** | 0.05 | 0.525 | 1.0 |
| **pH** | 2 | 5 | 8 |
| **CT (min)** | 5 | 32.5 | 60 |

**Table S2:** **The properties of bentonite obtained from BET analysis**

| **Property** | **Value** |
| --- | --- |
| Specific gravity | 0.002 |
| BET surface area, m^2^/g | 87.1779 |
| Particle size, nm^3^ | 34.378 |
| Bulk density g/cm^3^ | 2.00 |
| Pore volume, cm^3^/g | 0.046441 |
| Pore diameter, Å |  |

**Table S3: Experimental plan and the percentage Cr (VI) AN for bentonite**

| A: pH | B: AD | C: ICC | D: CT | % AN |
| --- | --- | --- | --- | --- |
| 4 | 0.1 | 200 | 5 | 72.34 |
| 4 | 0.1 | 50 | 5 | 65.13 |
| 4 | 0.55 | 125 | 32.5 | 69.5 |
| 5 | 0.55 | 125 | 32.5 | 62.25 |
| 4 | 1 | 200 | 60 | 86.92 |
| 5 | 0.55 | 200 | 32.5 | 73.4 |
| 5 | 0.55 | 125 | 32.5 | 62.8 |
| 4 | 0.1 | 50 | 60 | 65.2 |
| 5 | 0.55 | 125 | 32.5 | 61.3 |
| 5 | 0.55 | 125 | 32.5 | 63.6 |
| 5 | 0.55 | 125 | 5 | 59.3 |
| 5 | 0.55 | 125 | 32.5 | 61.95 |
| 6 | 0.1 | 200 | 60 | 68.49 |
| 6 | 1 | 50 | 60 | 60.2 |
| 6 | 0.1 | 50 | 5 | 54.6 |
| 6 | 0.1 | 200 | 5 | 65.6 |
| 5 | 0.55 | 125 | 32.5 | 61.5 |
| 5 | 0.55 | 125 | 60 | 64.4 |
| 6 | 1 | 200 | 5 | 71.3 |
| 4 | 1 | 200 | 5 | 82.4 |
| 6 | 1 | 50 | 5 | 58.2 |
| 4 | 1 | 50 | 60 | 69.95 |
| 6 | 0.1 | 50 | 60 | 53.56 |
| 5 | 1 | 125 | 32.5 | 63.57 |
| 5 | 0.55 | 50 | 32.5 | 63.34 |
| 4 | 1 | 50 | 5 | 71.2 |
| 4 | 0.1 | 200 | 60 | 75.6 |
| 5 | 0.1 | 125 | 32.5 | 57.4 |
| 6 | 0.55 | 125 | 32.5 | 57.98 |
| 6 | 1 | 200 | 60 | 80.62 |

**Table S4: The evaluation isotherm parameters for the removal of chromium**

| **Model** | **Constants** | **Values** | **Equation** |
| --- | --- | --- | --- |
| **Freundlich** | n | 0.406 |  |
|  | K_F_ (mg^1−n^L^n^/g) | 0.0035 | $qe=0.0035*{Ce}^{2.46}$ |
|  | R^2^ | 0.846 |  |
| **Langmuir** | q_max_ (mg/g) | 25.17 | $qe=\frac{0.0044*Ce}{1+0.0133*Ce}$ |
|  | K_L_ (L/mg) | 0.0133 |  |
|  | R_L_ | 0.27 |  |
|  | R^2^ | 0.928 |  |
| **D-R** | $\beta$ | -0.0026 |  |
|  | q_m_ (mg/g) | 146.93 |  |
|  | E (kJ/mol^2^) | 0.0096 | $qe=12.327*e^{(0.00026*\varepsilon^{2})}$ |
|  | R^2^ | 0.73 |  |
| **Temkin** | B_T_ | 28.36 |  |
|  | A_T_ | 0.037 | $qe=$88.8*ln(8.913*Ce) |
|  | R^2^ | 0.68 |  |

**List of Figures**

**
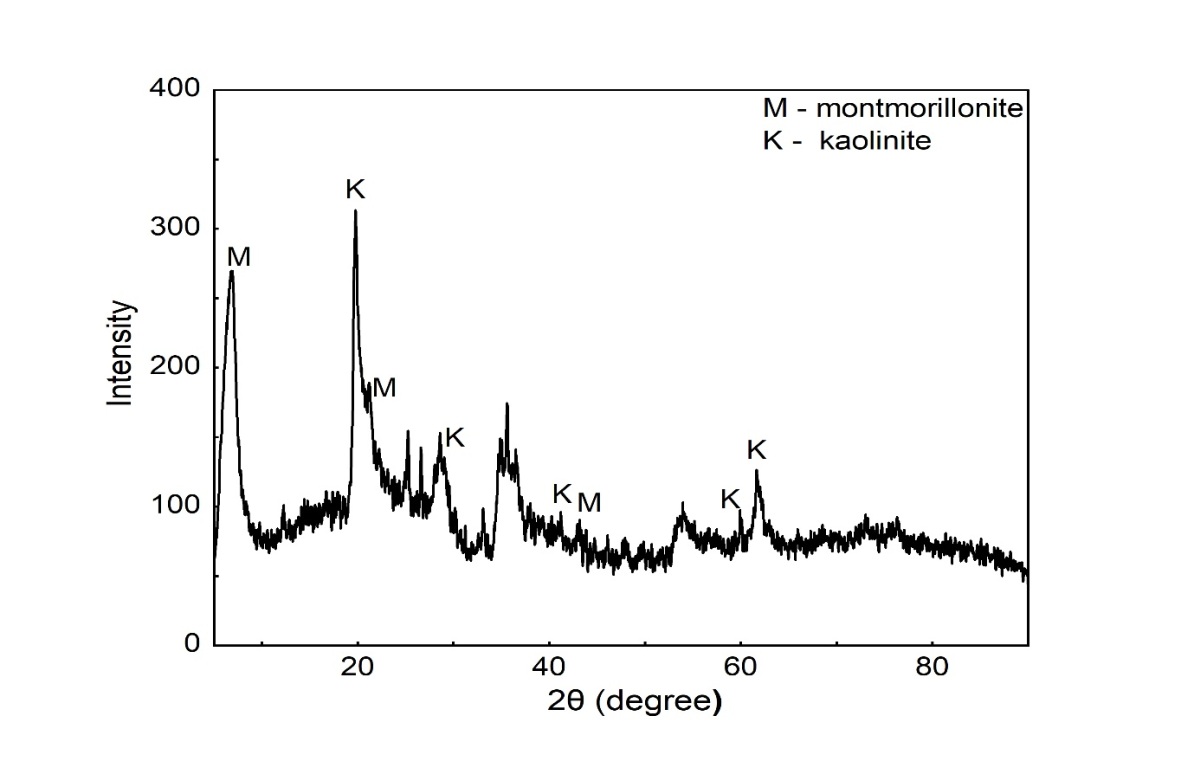
**

**Fig. S1: XRD pattern obtained for the AB bentonite**


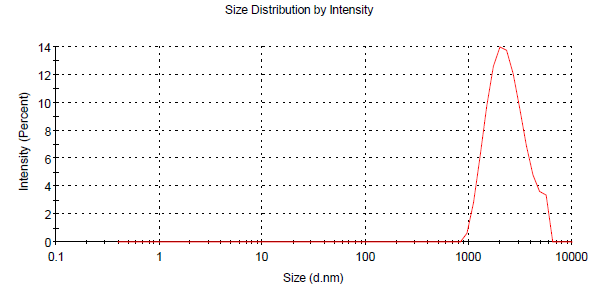


**Fig. S2: Particle size distribution of the Bentonite**

| 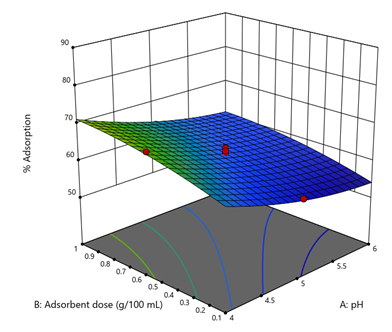 | 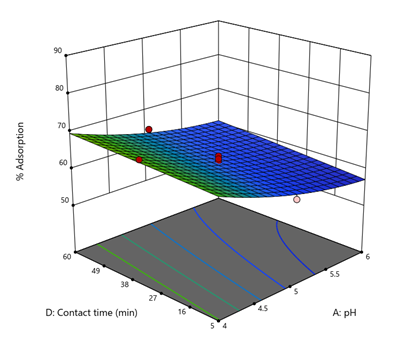 |
| --- | --- |
| 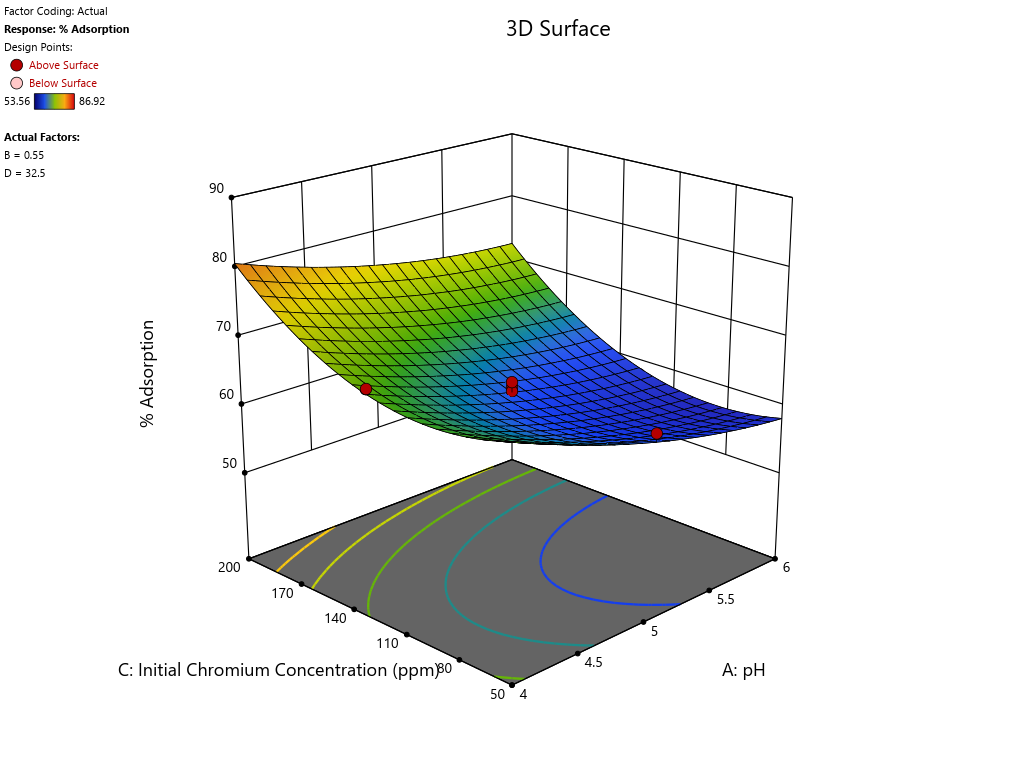 | 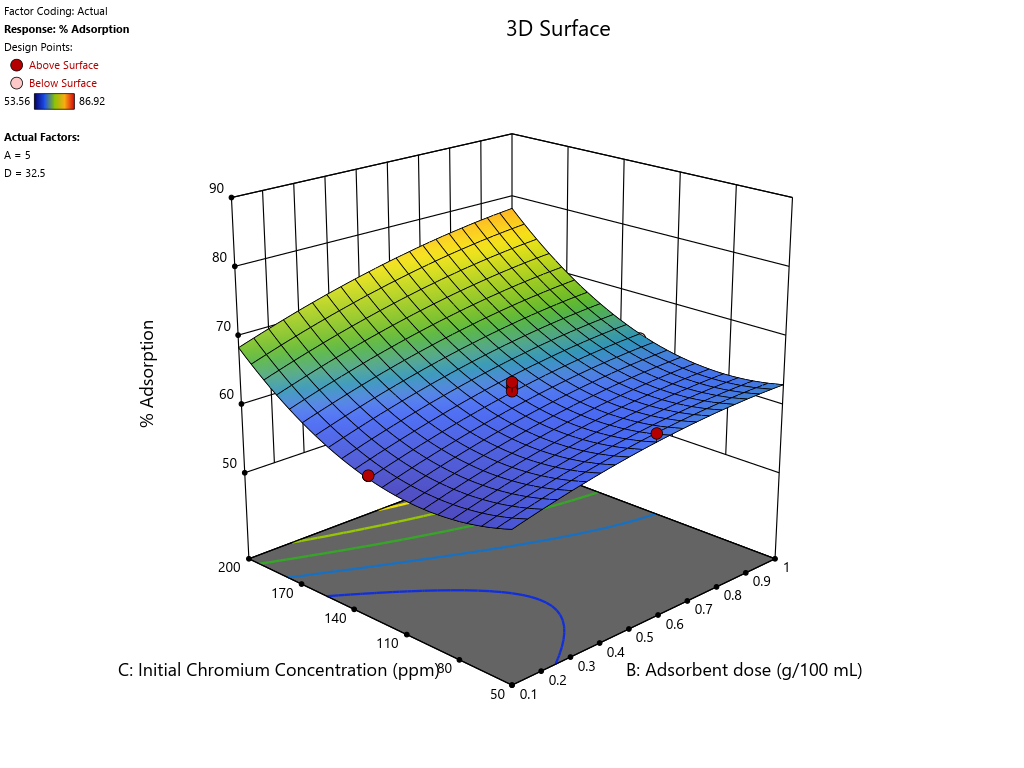 |
| 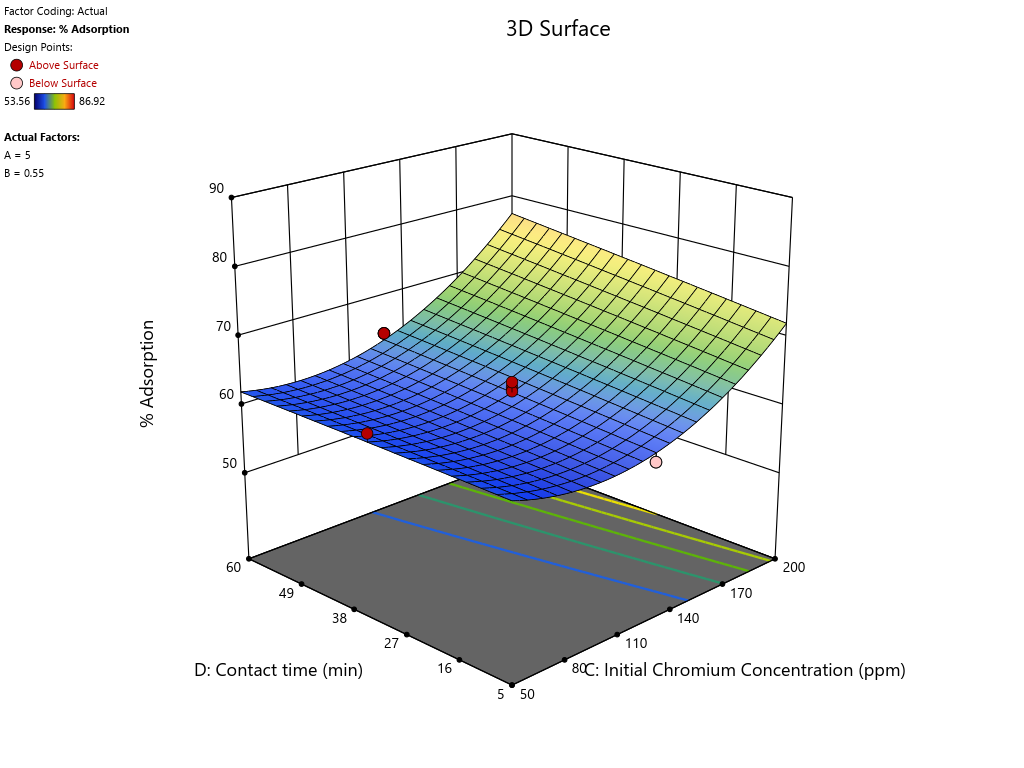 | 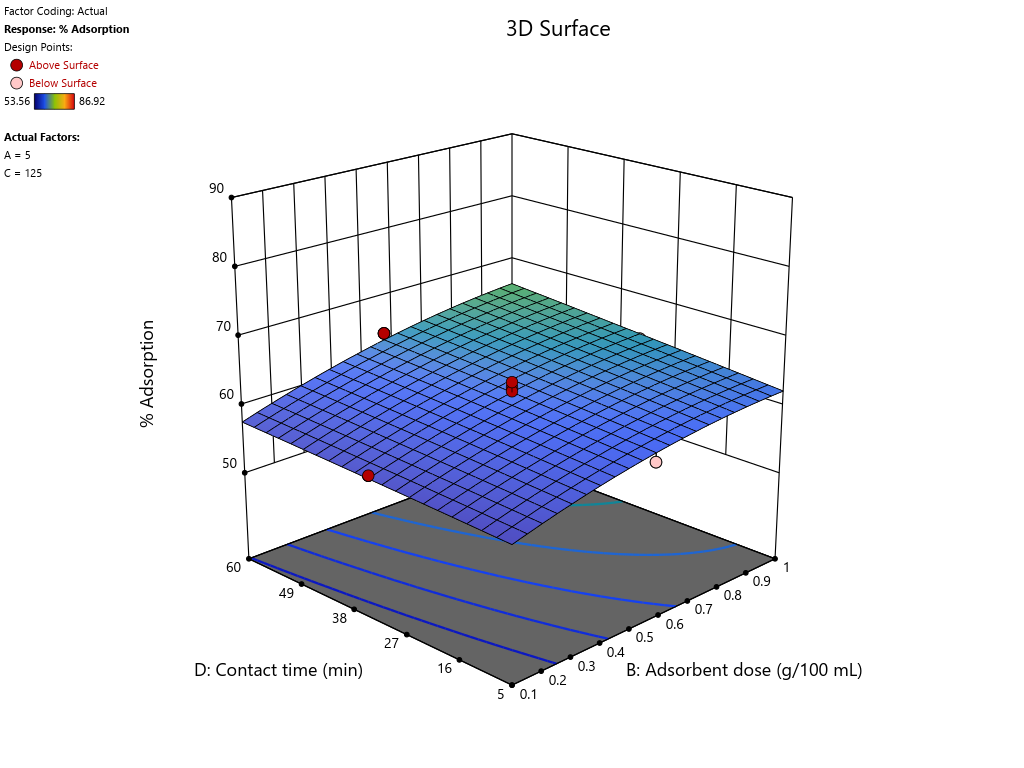 |

**Fig. S3: 3-D Response surface plots with respect to various parameters**

|  |
| --- |
| 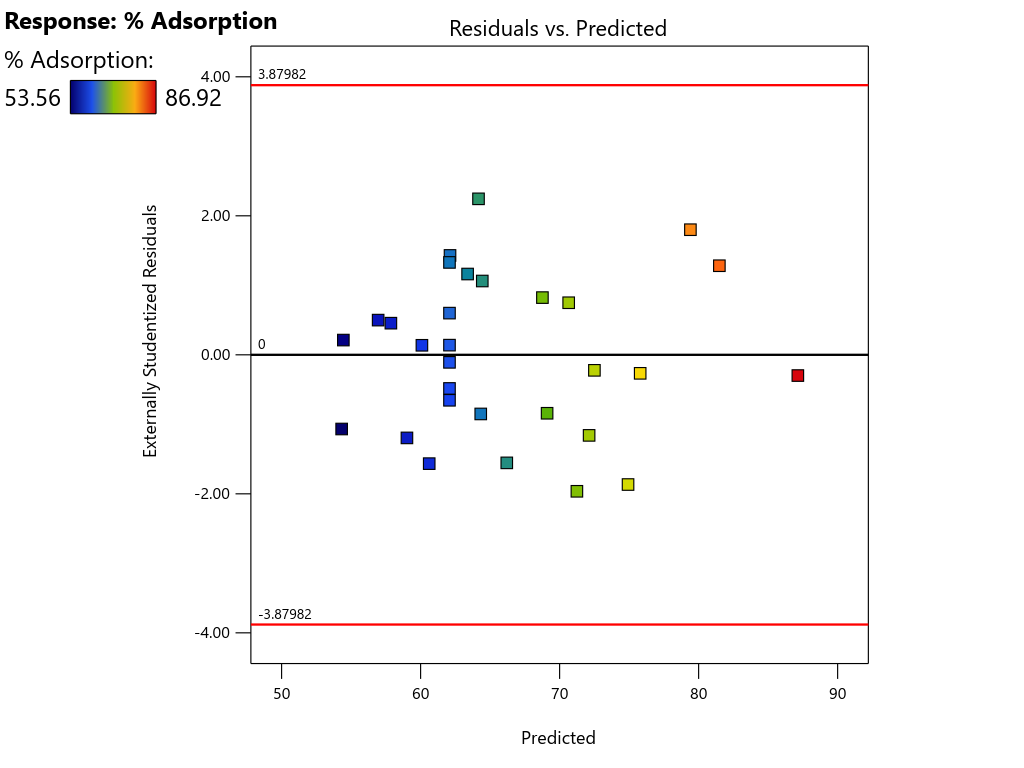 |
| **(a)** |
| 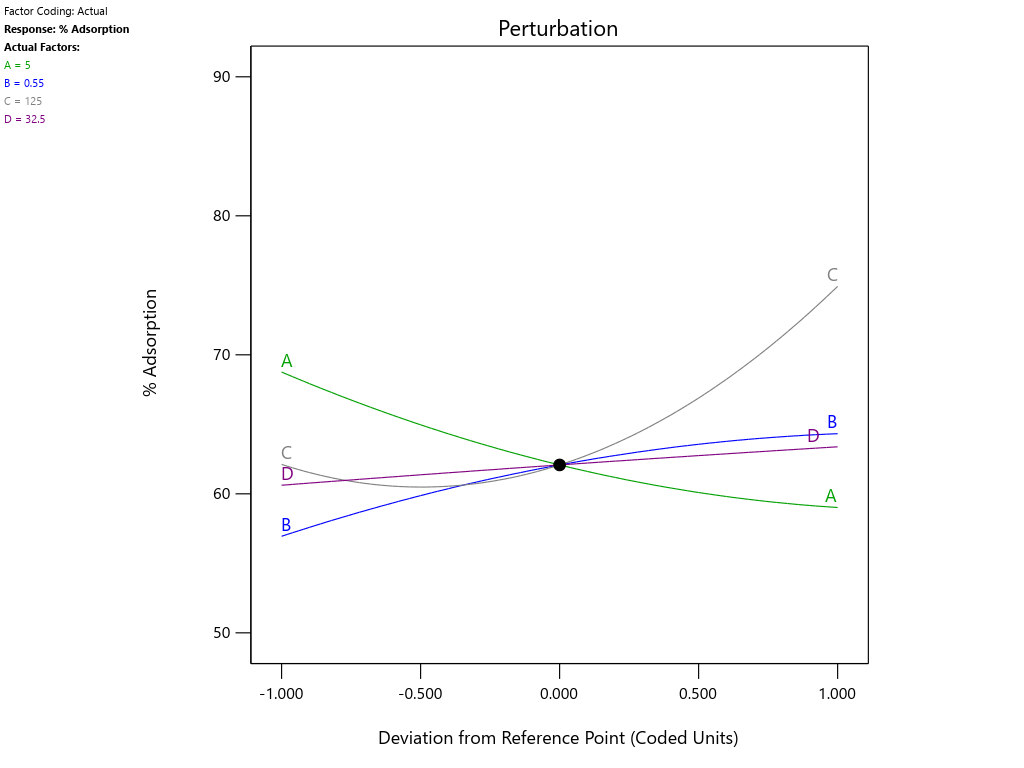 |
| **(b)** |
| 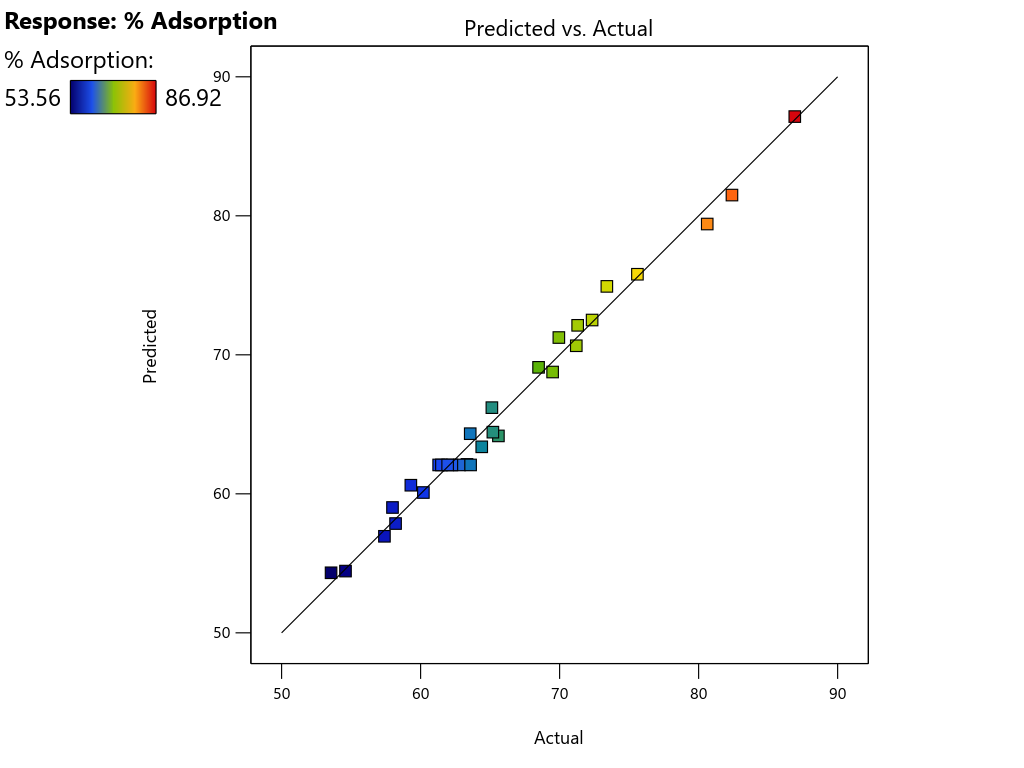 |
| **(c)** |
| 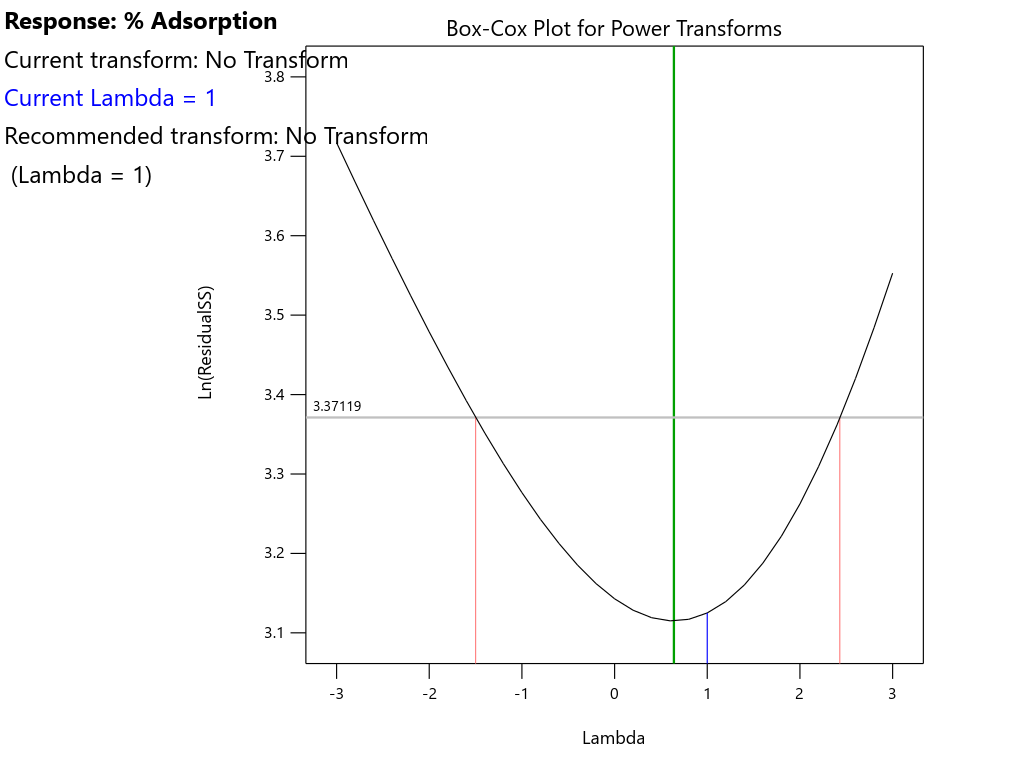 |
| **(d)** |

**Fig. S4: Diagnostic plots generated during ANOVA (a) Residual (b) perturbation (c) predicted vs actual (d) Box-Cox transformation plots**

| 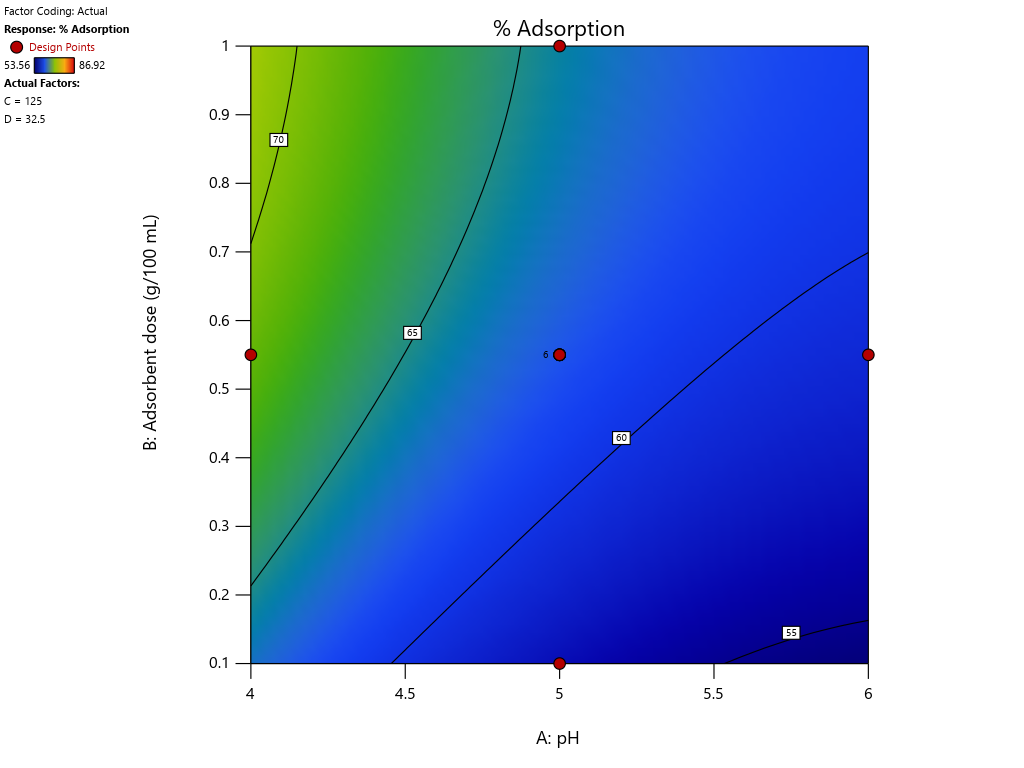 | 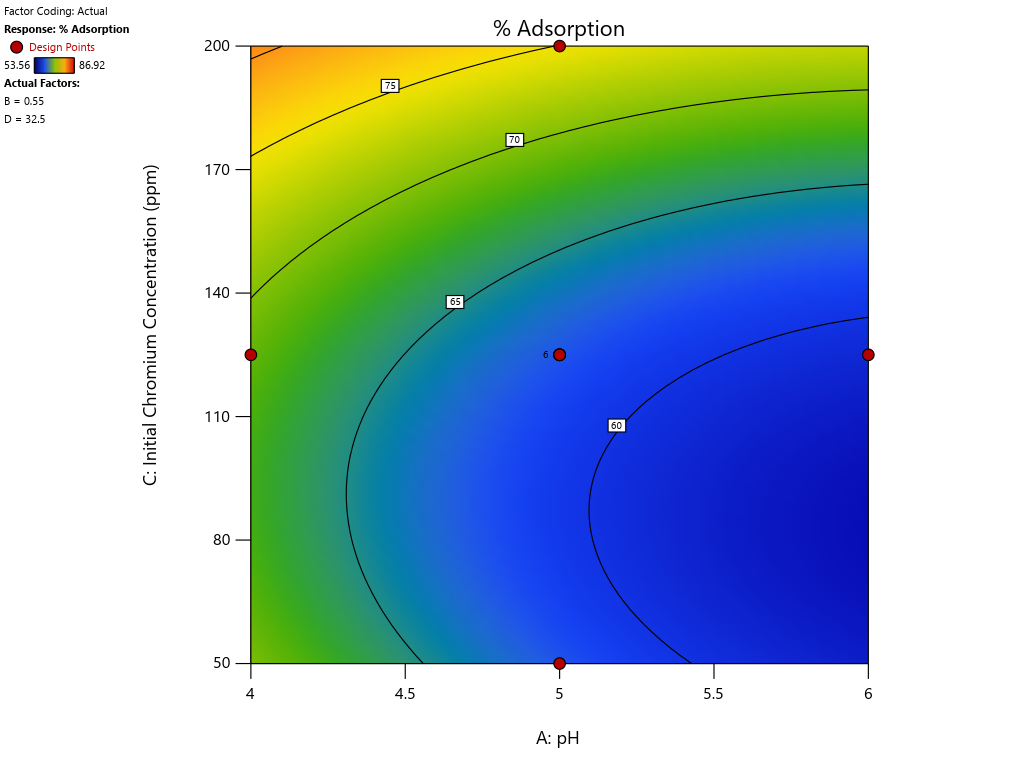 |
| --- | --- |
| 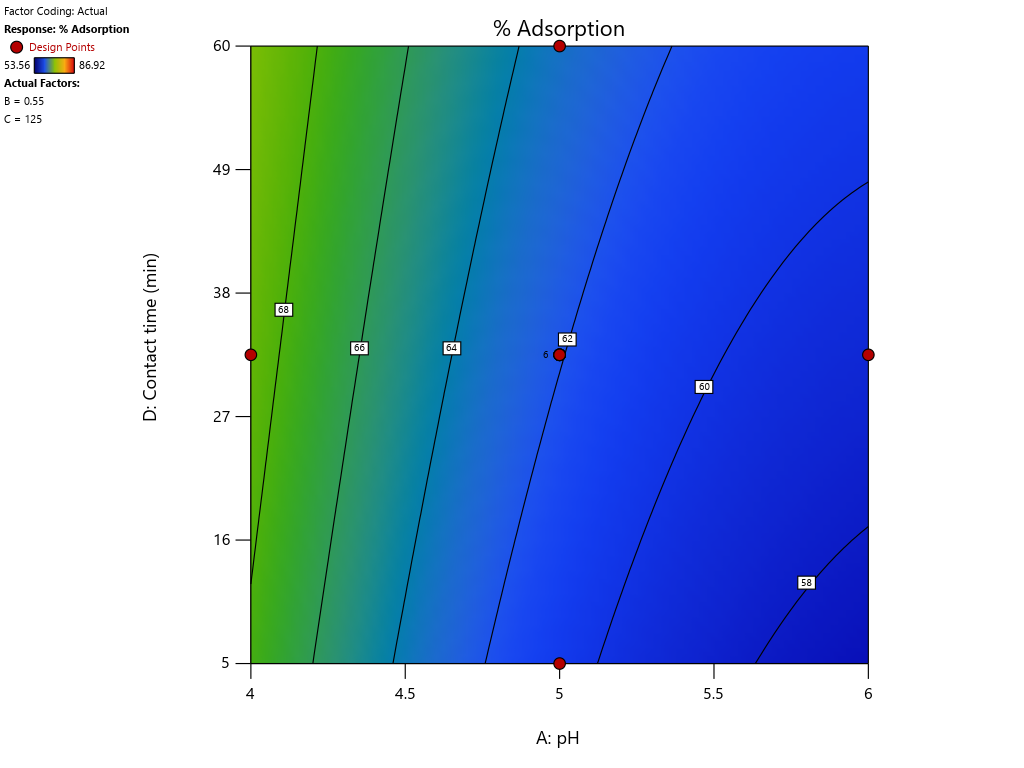 | 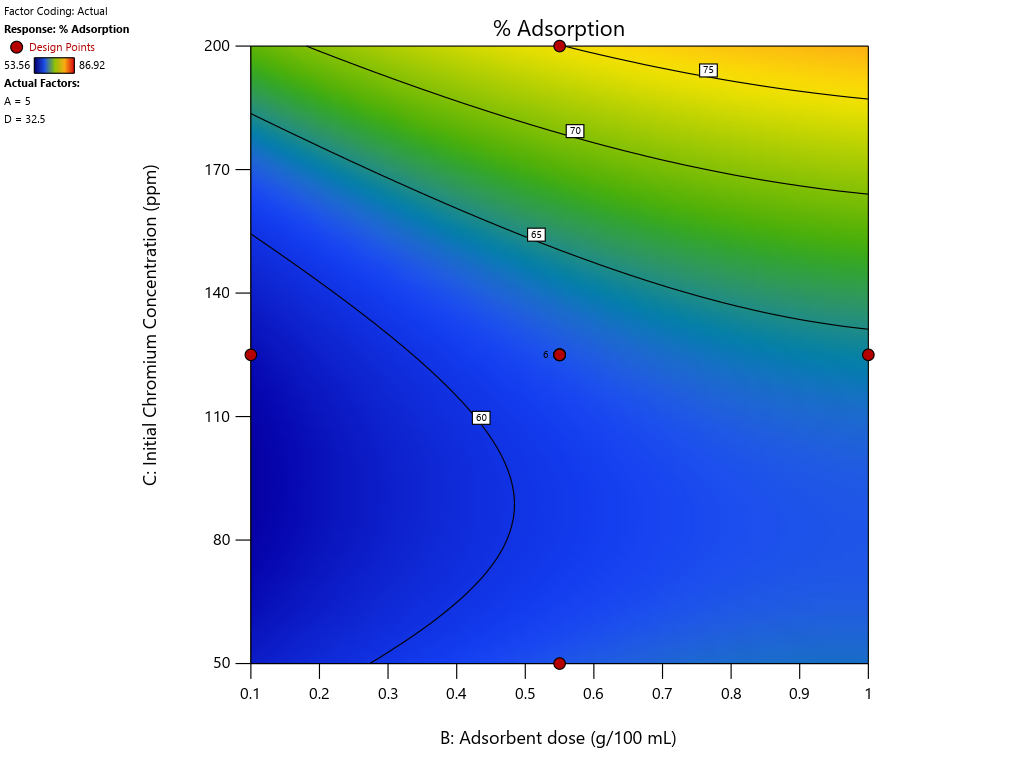 |
| 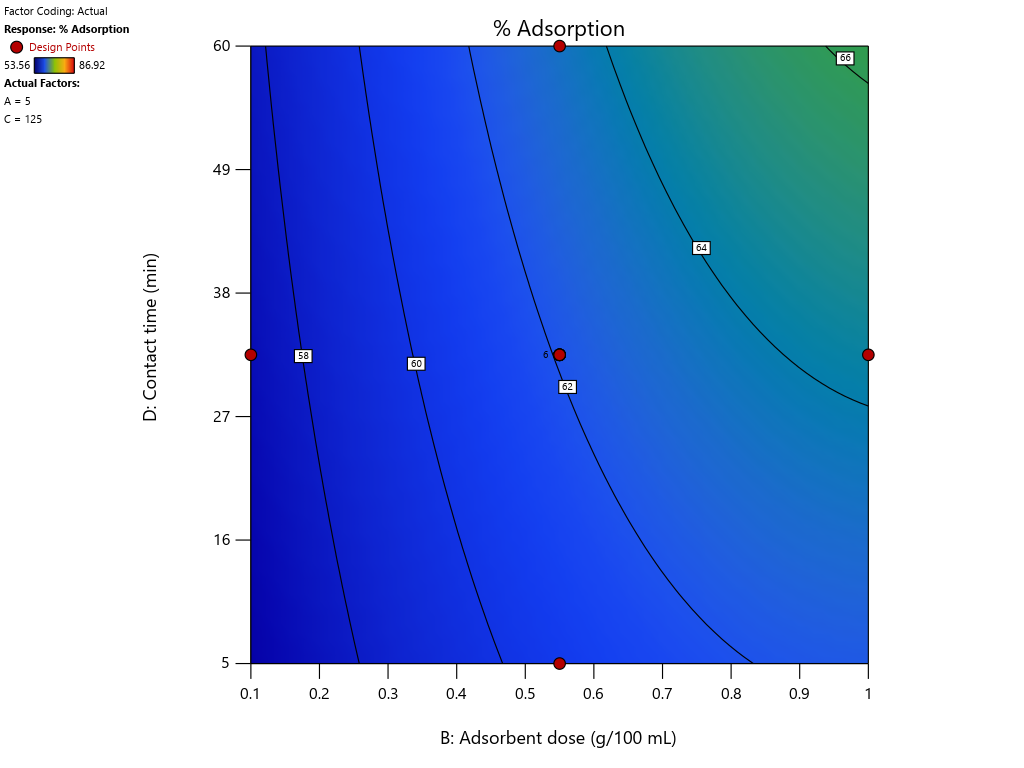 | 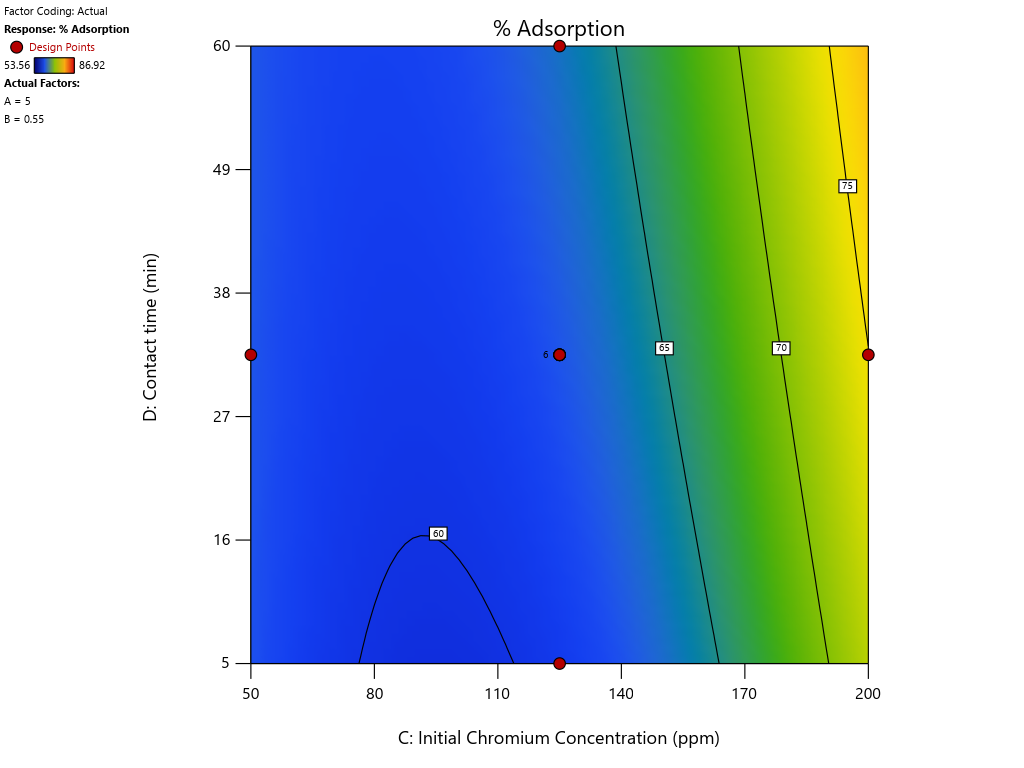 |

**Fig. S5: Contour plots on effect of independent variables on the AN capacity**

**Fig S6: The influence of temperature on removal of chromium.**

**Fig. S7: The effect of coexisting ions on the removal of chromium.**

**Fig. S8: Reusability of used bentonite over various cycles**


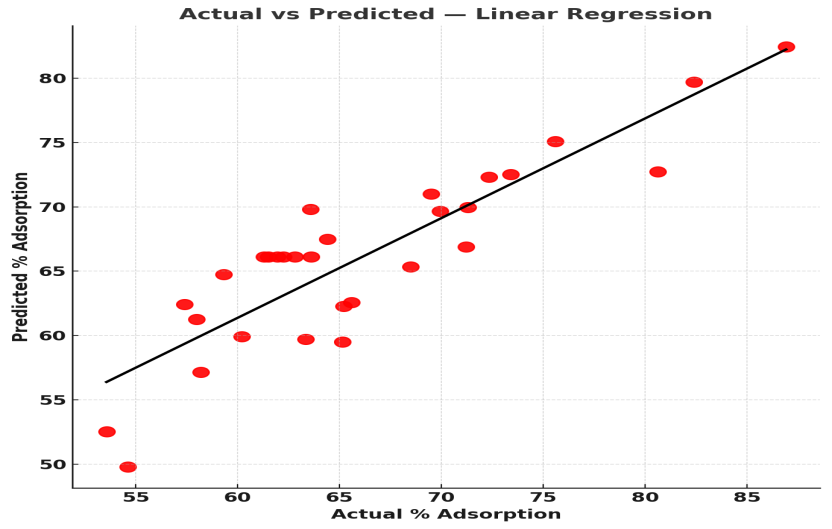


**Fig. S9 (a): Actual v/s predicted values of LR model for pollutant removal study**


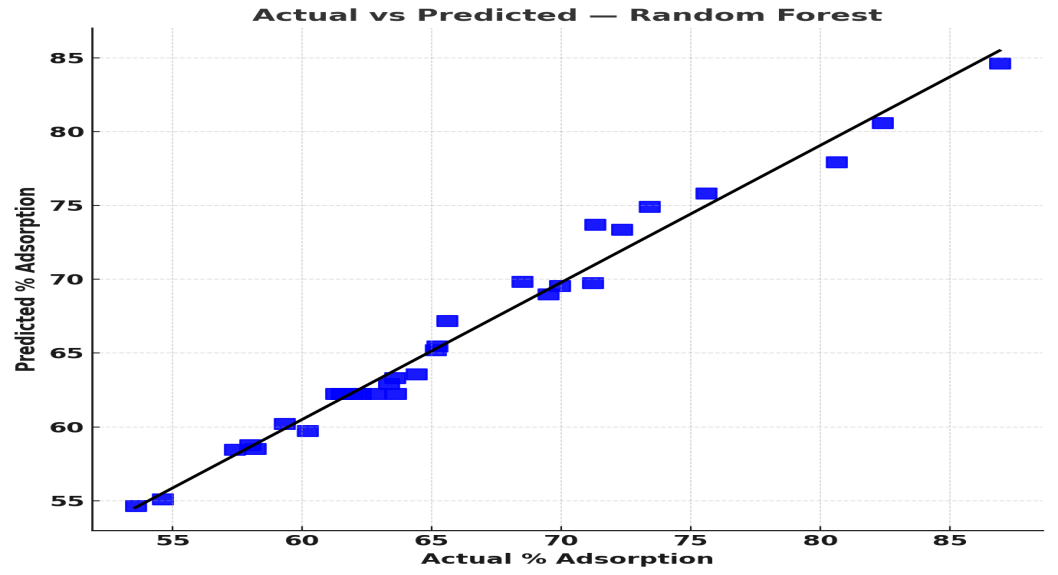


**Fig. S9 (b): Actual v/s predicted of RF model for pollutant removal study**


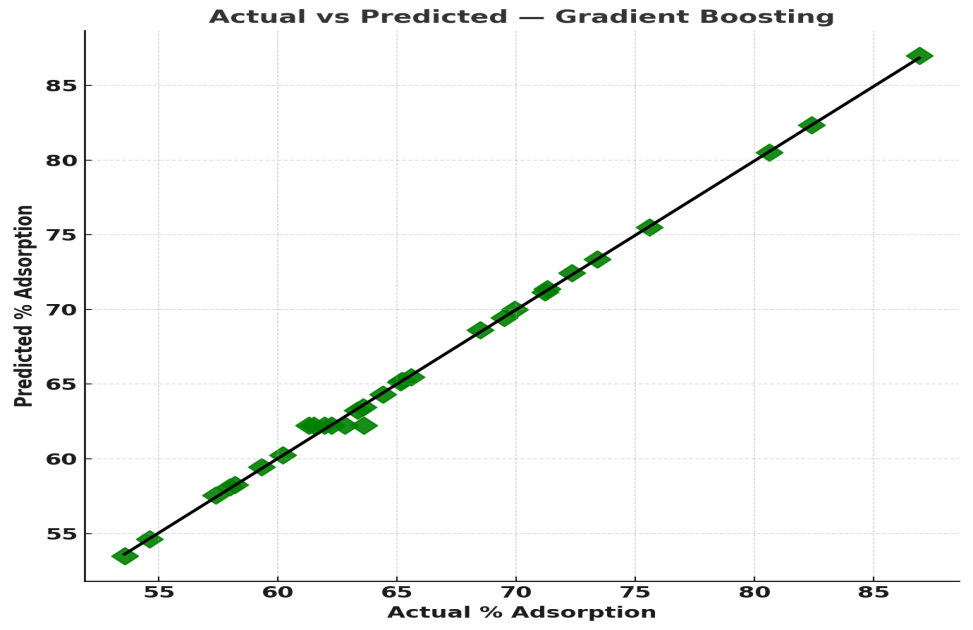


**Fig. S9 (c): Actual v/s predicted of GB model for pollutant removal**


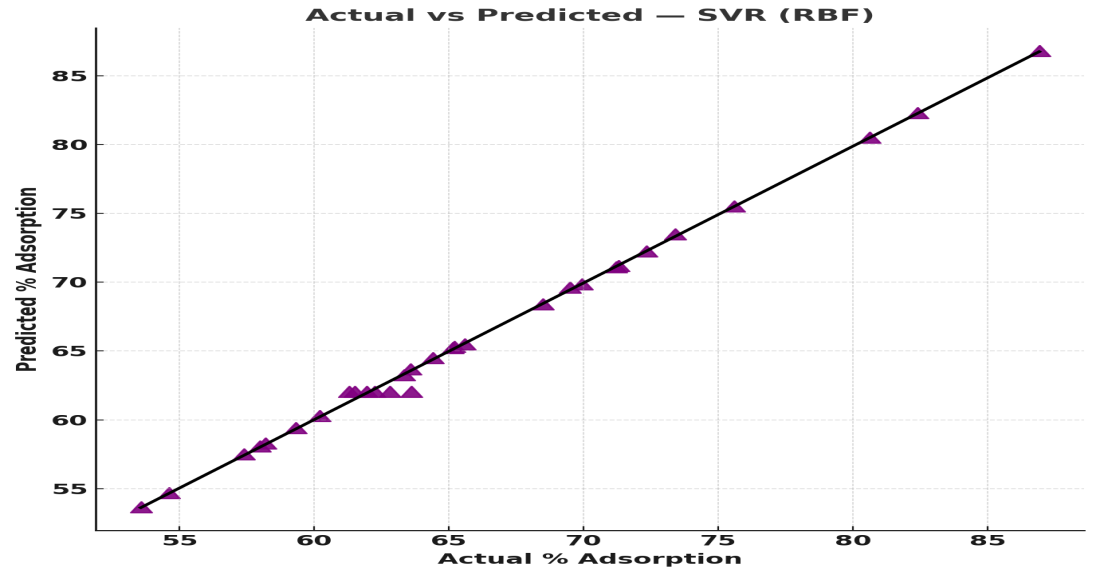


**Fig. S9 (d): Actual v/s predicted of SVR (RBF) model for pollutant removal study**


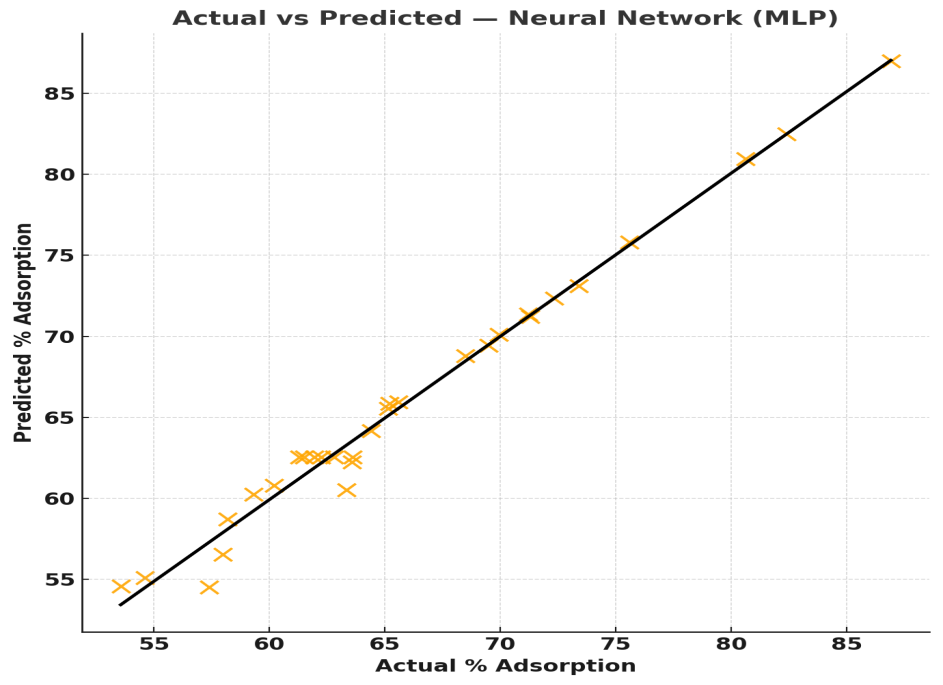


**Fig. S9 (e): Actual v/s predicted of ANN model for pollutant removal**


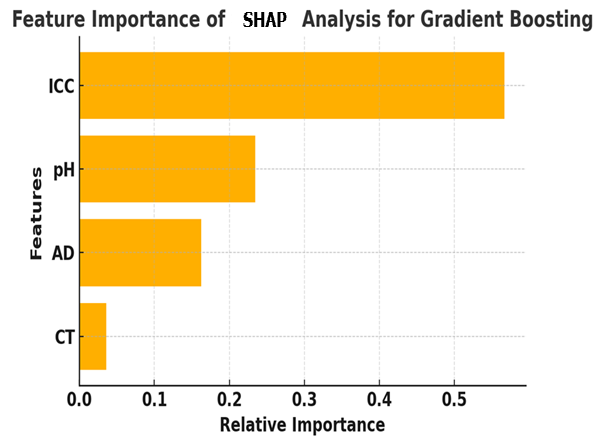


**Fig. S10: Feature importance of SHAP analysis for Gradient Boosting**

| 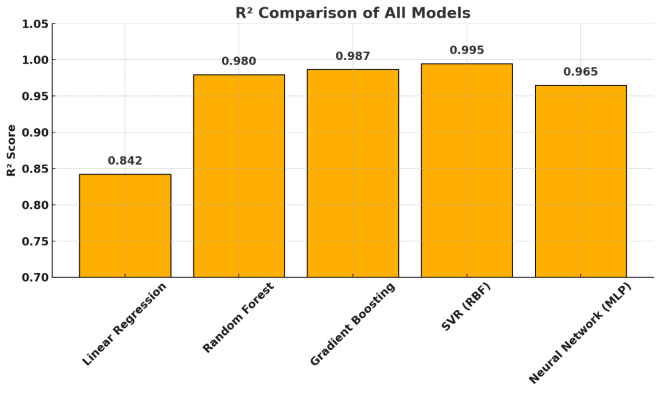 |
| --- |
| **(a)** |
| 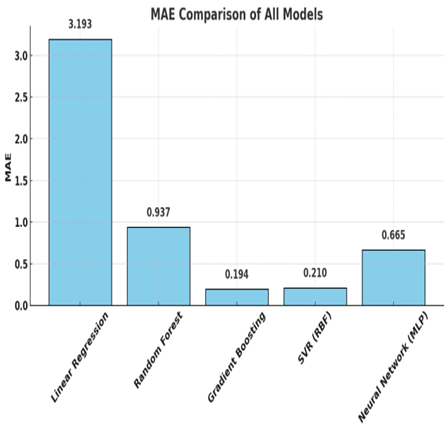 |
| **(b)** |
| 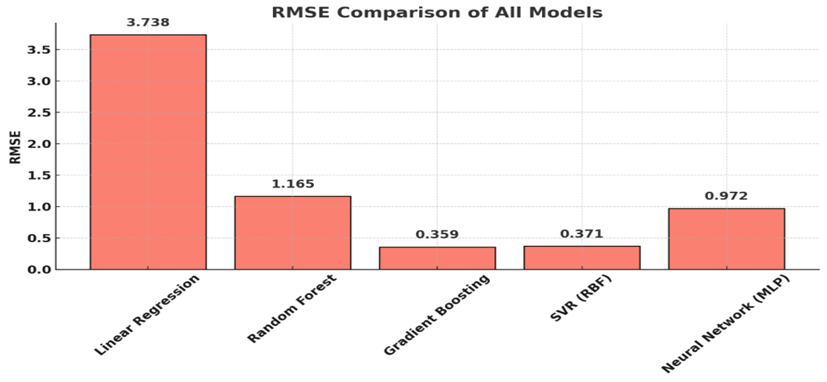 |
| **(c)** |

**Fig. S11 (a-c):** $\mathbf{R}^{\mathbf{2}}$**, MAE, and RMSE prediction models for AN property of bentonite**

**
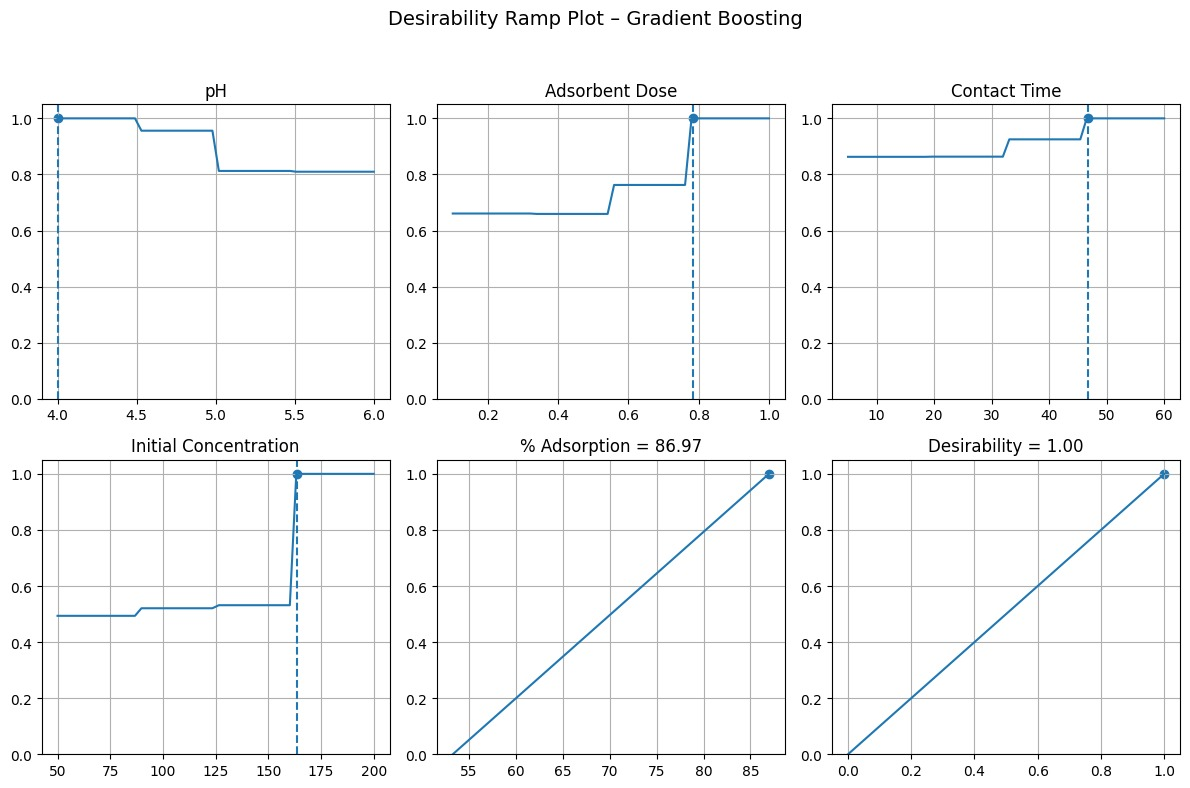
**

**Fig. S12 (a): Desirability Ramp plot for GB prediction models for AN property of bentonite**


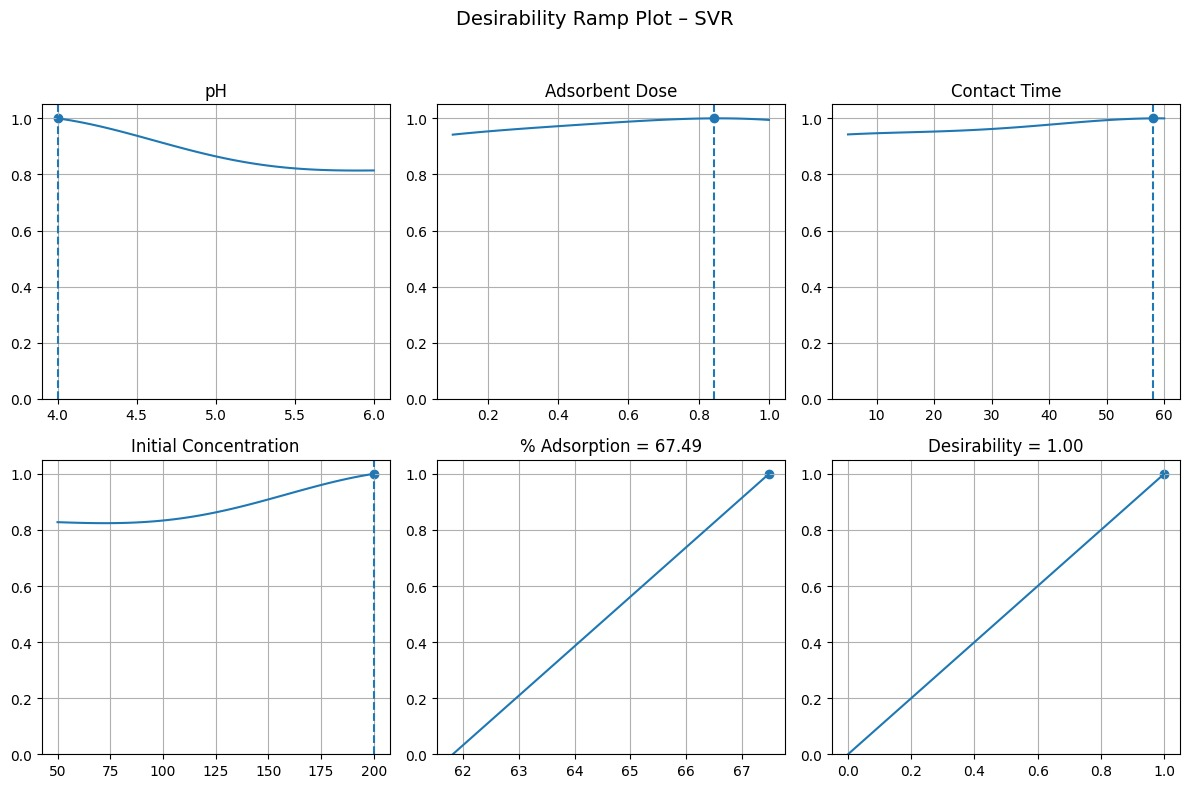


**Fig. S12 (b): Desirability Ramp plot for SVR prediction models for AN property of bentonite**


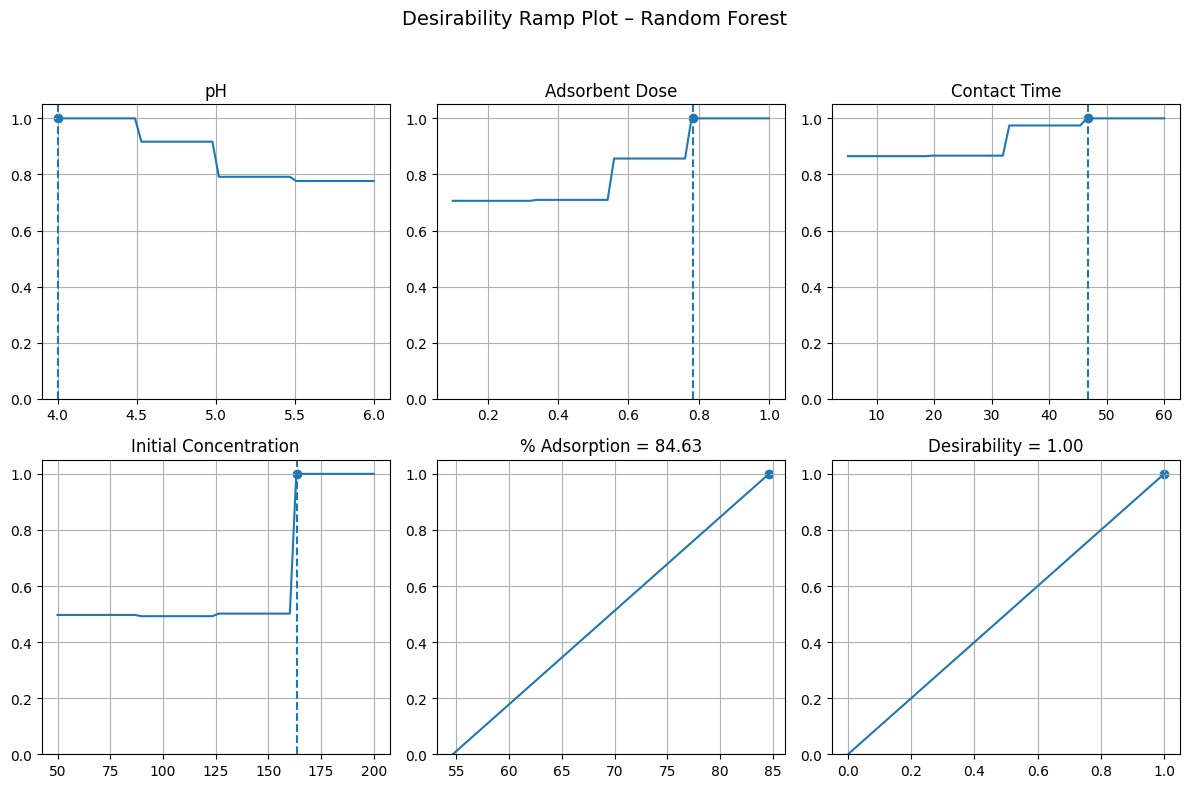


**Fig. S12 (c): Desirability Ramp plot for RF prediction models for AN property of bentonite**


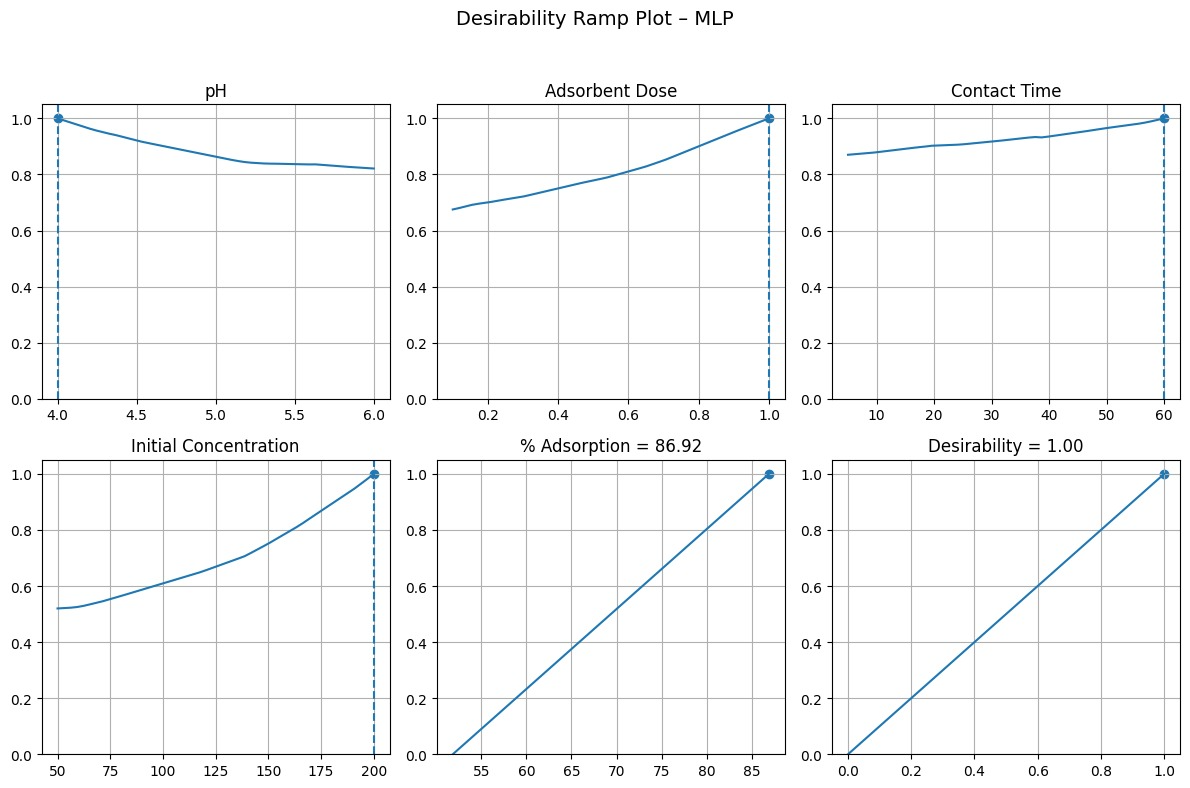


**Fig. S12 (d): Desirability Ramp plot for RF prediction models for MLP property of bentonite**


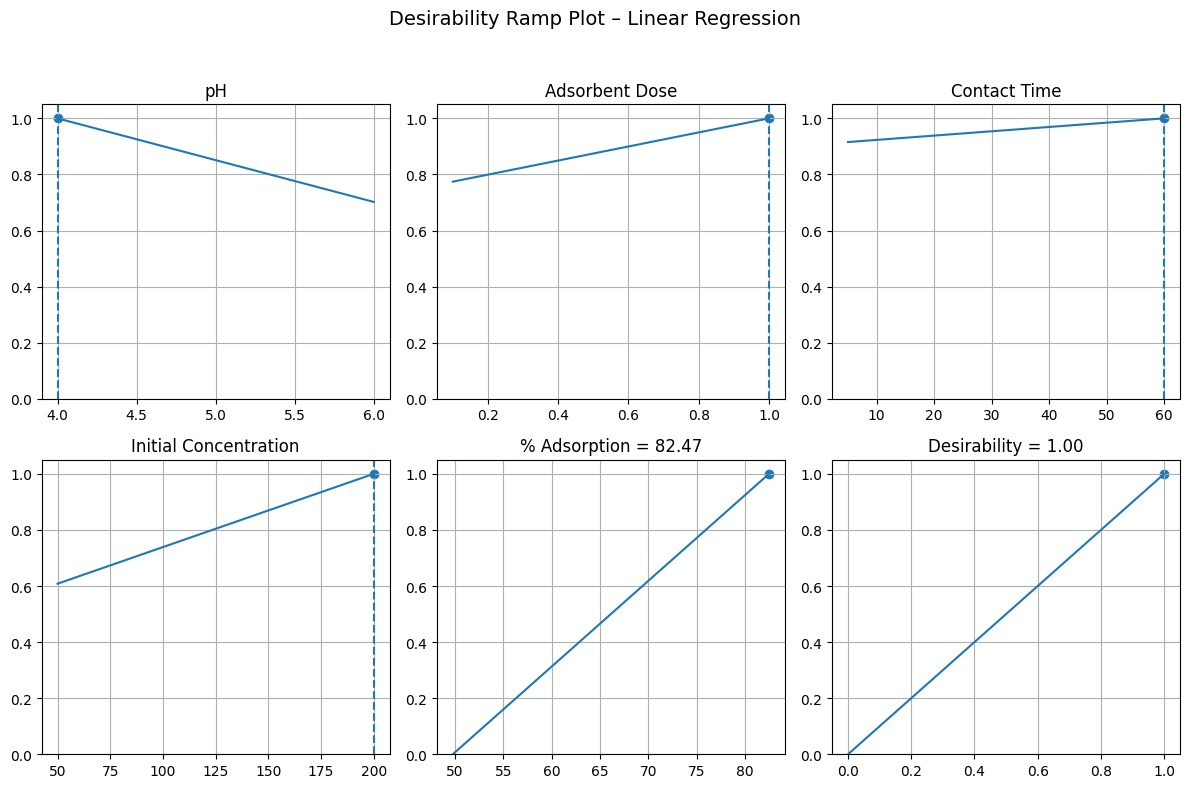


**Fig. S12 (e): Desirability Ramp plot for LR prediction models for AN property of bentonite**

***
